# Nutritional Adequacy of Animal-Based and Plant-Based Asian Diets for Chronic Kidney Disease Patients: A Modeling Study

**DOI:** 10.3390/nu13103341

**Published:** 2021-09-24

**Authors:** Ban-Hock Khor, Dina A. Tallman, Tilakavati Karupaiah, Pramod Khosla, Maria Chan, Joel D. Kopple

**Affiliations:** 1 Faculty of Food Science and Nutrition, Universiti Malaysia Sabah, Kota Kinabalu 56000, Sabah, Malaysia; khorbanhock@gmail.com; 2 Department of Nutrition and Food Science, Wayne State University, Detroit, MI 48202, USA; dina.tallman@wayne.edu (D.A.T.); aa0987@wayne.edu (P.K.); 3 US Food and Drug Administration, Detroit, MI 48207, USA; 4 School of Biosciences, Faculty of Health and Medical Sciences, Taylor’s University, Subang Jaya 47500, Selangor, Malaysia; tilly_karu@yahoo.co.uk; 5 Departments of Renal Medicine and Nutrition and Dietetics, The St. George Hospital, Kogarah, NSW 2217, Australia; Maria.Chan@health.nsw.gov.au; 6 Harbor-UCLA Medical Center, The Lundquist Institute, Torrance, CA 90502, USA; 7 David Geffen School of Medicine at UCLA and UCLA Fielding School of Public Health, Los Angeles, CA 90095, USA

**Keywords:** chronic kidney disease, plant-based diet, vegetarian diet, low protein diet, amino acids, micronutrients, Asian nutrition

## Abstract

Plant-based low protein diets (LPDs) have gained popularity for managing chronic kidney disease (CKD) patients. The nutritional adequacy of these and other LPDs prescribed for CKD patients have not been carefully examined. This study assessed the nutrient composition of such LPDs and moderately high protein diets (MHPDs) that might be prescribed for patients in the Asia Pacific region with CKD who are not dialyzed or undergoing maintenance dialysis. Conventional diets containing at least 50% animal-based proteins and plant-based diets were also planned with protein prescriptions of 0.5 to 0.8 g/kg/day and MHPDs with protein prescriptions of 1.0 to 1.2 g/kg/day. Plant-based, lacto-, ovo-, and lacto-ovo-vegetarian and vegan LPDs and MHPDs were planned by replacing some or all of the animal proteins from the conventional diet. With 0.5 g protein/kg/day, all diets were below the Recommended Dietary Allowances (RDA) for at least one essential amino acid (EAA). At a protein prescription of 0.6 g/kg/day, only the conventional LPD met the RDA for all EAAs. This deficiency with the plant-based LPDs persisted even with several plant food substitutions. With a protein prescription ≥0.7 g/kg/day, all the plant-based and vegetarian LPDs provided the RDA for all EAA. The plant-based and vegetarian diets also contained relatively greater potassium, phosphorus, and calcium content but lower long-chain *n*-3 polyunsaturated fatty acids and vitamin B-12 than the conventional diet. Other essential micronutrients were commonly below the RDA even at higher protein intakes. The low contents of some essential micronutrients were found in both animal-based and plant-based diets. Prescription of all LPDs for CKD patients, especially plant-based and vegetarian LPDs, requires careful planning to ensure the adequacy of all nutrients, particularly essential amino acids. Consideration should be given to supplementing all animal-based and plant-based LPDs and MHPDs with multivitamins and certain trace elements.

## 1. Introduction

Chronic kidney disease (CKD) is a global health concern. A recent systematic analysis estimates that 9.1% of the worldwide adult population is affected by CKD, and almost a third of people with CKD live in China and India [1]. A low protein diet (LPD) providing 0.6 to 0.8 g protein per kg body weight has been recommended for patients with non-dialysis dependent CKD stages 3 to 5 since protein restriction engenders a reduction of proteinuria and decreases the generation of protein-derived toxins, including acid, other metabolites and phosphate [2,3]. Contrarily, a greater amount of protein (1.0–1.2 g/kg) is recommended for CKD patients undergoing maintenance dialysis to compensate for the increase in protein catabolism as well as protein, peptide, and amino acid losses into the dialysate that occur during dialysis treatments [3,4].

Recently, vegetarian and plant-based diets have been proposed as part of healthier and more environmentally sustainable eating patterns. Although the terms “vegetarian diet” and “plant-based diet” are often used interchangeably, they represent two different eating patterns. Vegetarian diets eliminate flesh foods, including meat, poultry, wild game, and seafood, while their products, such as eggs and milk, may be excluded [5]. Contrarily, plant-based diets emphasize consuming foods mainly from plants, such as fruit, vegetables, nuts, healthy oils, whole grains, and legumes, with the inclusion of small quantities of animal-based food sources, including milk, eggs, meat, and fish [6]. A growing body of epidemiological evidence supports the recommendation of plant-based and vegetarian diets to prevent and treat certain nutrition-related chronic diseases such as obesity, type 2 diabetes, hypertension, cardiovascular diseases, and some cancers [5,7]. A number of reports suggest that plant-based foods may be associated with favorable effects on CKD patients with regard to blood pressure, phosphorus burden, acid load, uremic toxins, inflammation, and oxidative stress [8,9,10,11,12,13,14]. However, these purported benefits have largely not yet been confirmed in randomized prospective controlled clinical trials with clinical outcomes as the key outcome measures.

Since plant proteins are more likely to contain lower amounts of certain essential amino acids (EAAs), especially lysine and the sulfur-containing amino acids [15], as well as lesser quantities of some micronutrients [5], it has been questioned as to whether these plant-based LPDs may be deficient in some essential nutrients. A few interventional studies from Italy [16,17] and Israel [18] did not describe an increased prevalence of protein-energy wasting or malnutrition in non-dialyzed CKD patients prescribed vegetarian LPDs ranging from 0.7 to 0.75 g protein per kg body weight. However, the assessment of nutritional status performed in these studies only included body weight and various serum protein markers, whereas the patients’ amino acid, vitamin, mineral, and trace element status were not evaluated. These latter reports also did not carefully track the actual nutrient intake of the CKD patients that were studied. On the other hand, an observational study in Taiwan reported that lacto-ovo vegetarians with CKD had a lower dietary energy and protein intakes and lower body mass index than their omnivore counterparts [19]. The self-reported dietary protein intake of these latter patients ranged from 0.79 to 0.92 g per kg body weight. Thus, although diets providing as low as 0.6 g protein/kg/day are recommended, there is very little information concerning the adequacy of essential nutrients, including the EAAs, of vegetarian or plant-based LPDs providing 0.5 to 0.6 g protein per kg body weight.

Vegetarianism has a long tradition in Indian cultures due at least partly to religious interdictions against taking living creatures’ lives [20]. At present, about 40% of India’s population traditionally adheres to vegetarian diets, which primarily include fermented milk (i.e., curds), lentils, and fresh sprouting seeds such as green gram/mung bean as the protein source [20]. Similarly, vegetarianism in China is also encouraged by the practice of Buddhism and Daoism, and the Chinese vegetarian diet generally includes soybean products, such as soybean curd (*tofu*) and textured vegetable protein as protein sources [21]. The nature and nutritional adequacy of vegetarian or plant-based diets prescribed to CKD patients from these two cultural backgrounds have not been carefully examined, even though these two countries have, by far, the largest populations in the world. In addition, the literature on vegetarian and plant-based diets for CKD management is mainly proposed for Western societies, and little has been published about its applicability in Asian cultures. Therefore, the present study examined the nutritional adequacy of EAA and micronutrients of plant-based and vegetarian diets for Asian CKD patients, primarily of Chinese or Indian cultural backgrounds. Since there is also little published information concerning the essential nutrient content of animal-based LPDs prescribed for CKD patients, we analyzed the essential nutrients in the more conventional animal-based LPDs and moderately high protein diets (MHPDs) that might be prescribed to Asian CKD patients.

## 2. Materials and Methods

### 2.1. Planning of a Conventional Menu

As a comparator reference diet, we designed a conventional 3-day animal-based menu using 70 kg as the reference body weight for an adult (Table 1). The LPDs consisted of four protein prescriptions, providing 0.5, 0.6, 0.7, and 0.8 g protein per kg body weight. At least 50% of the protein was high biological value protein derived from animal-based food sources [22]. MHPDs providing 1.0, 1.1, and 1.2 g protein per kg body weight which are often prescribed for maintenance hemodialysis and peritoneal dialysis patients were also analyzed [2,3]. The energy prescription was set at 2100 kcal for all diets, based on 30 kcal per kg body weight. Total carbohydrates contributed 54 to 58% of energy (284–305 g) across all protein prescriptions, while the proportion of total fat in the diets ranged from 30 to 35% of energy (70–82 g).

We then determined the exchange or serving size for each food group based on the macronutrient distribution across protein prescriptions. All meal plans were fixed to provide three exchanges of fruit and four servings of non-starchy vegetables. For LPDs, there were eight exchanges of cereals or cereal products and 14 exchanges of fat. Variation for the number of protein food exchanges ranged from two to five exchanges with three to five exchanges of sugar. For MHPDs, there were 11 exchanges of cereals or cereal products. The protein foods varied in providing six to eight exchanges with 11 to 13 exchanges of fat.

### 2.2. Reference Menus

The approach to menu construction was centered on typical dietary patterns of Indian and Southern Chinese ethnic groups by reference to the patient education materials and the menus designed for patients with CKD from North Indian (Indraprastha Apollo Hospital, New Delhi, India), South Indian (Apollo Hospital Chennai, Tamil Nadu, India), and southern China (Guangzhou Red Cross Hospital, Guangzhou, China) hospitals These menus were obtained via personal communications with the dietitians (Anita Jatana, Apollo Hospital, New Delhi, India and Daphnee DK, Apollo Hospital, Chennai, Tamil Nadu, India) and a nephrologist (Prof. Dr. Tan Rong Shao, Guangzhou Red Cross Hospital, Guangzhou, China) living and working in these areas.

The cereals and cereal products group consisted primarily of rice, which is the staple food for Asians. Other cereal products on the menu included *dosa*, biscuits, and rice noodles. We included fruits and vegetables common to the Asian region, such as papaya, banana, pear, apple, mango, orange, okra, cucumber, eggplant, cabbage, and spinach. For LPDs, the preparation method for fish and poultry dishes was mainly deep-frying. Beverages such as tea, typically consumed with sugar in India, although not China was featured in the menu to promote adequacy towards the energy prescription without exceeding the protein limit.

### 2.3. Protein Food Substitutions in Plant-Based and Vegetarian Diets

We planned a menu for a plant-based and four vegetarian diets by substituting the animal-based protein in the conventional menu with plant-based protein such as soy products (i.e., *tofu* and *tempeh*), lentils (*dhal*), or beans. Other food groups on the plant-based and vegetarian menu remained the same as the conventional menu. Constructed menus are provided as supplementary material (Table S1). Menu details for each plant-based and vegetarian diet are as below:

Model 1: The plant-based diet was planned according to the principles of the plant-dominant (PLADO) diet proposed by Kalantar-Zadeh et al. [13]. This diet consisted of about 70% plant-based protein. For instance, a menu with a total protein content of 49 g/day would comprise 35 g protein from plant-based food and 14 g protein from animal-based food. Therefore, any animal-based food such as chicken, fish, or pork from the conventional menu was substituted with a plant-based food so that the animal-based food provided only 30% of total protein.

Model 2: A vegan diet that excluded all food derived from animals, including egg and dairy products. All animal-based protein was substituted with such plant-based protein as tofu, soybean milk, lentils, and chickpeas.

Model 3: An ovo-vegetarian diet that excluded all food derived from animals except for eggs. All animal-based protein, except eggs, was substituted with plant-based protein, and at least one exchange of egg (i.e., one whole egg or the whites from two eggs) was included in the daily diet. The maximum number of eggs included in the menu was three exchanges per day.

Model 4: A lacto-vegetarian diet that excluded all food derived from animals except for dairy products (such as milk, paneer, and curd). All animal-based protein, except dairy products, was substituted with plant-based protein, and at least one exchange of dairy products (i.e., 8 ounces or 240 mL milk) was included in the daily diet. The maximum number of dairy products included in the menu was two exchanges per day.

Model 5: A lacto-ovo vegetarian diet that excluded all food derived from animals except egg and dairy products. All animal-based protein was substituted with plant-based protein, and at least one exchange of egg and dairy products was included in the daily diet. The maximum number of egg and dairy products included in the menu was three and two exchanges per day, respectively.

### 2.4. Nutrient Analyses

The nutrient profile of all menus was analyzed using the Nutritionist Pro Software (Axxya Systems LLC, Redmond, WA, USA), which refers to the FoodData Central Database of the United States Department of Agriculture [23]. We omitted salt and seasonings in the menu construction and nutrient analysis because the use of these condiments varied by individual preferences and cooking practices, which did not reflect the nutrient composition differences caused by the substitution of animal-based protein with plant-based protein. Therefore, the sodium content of all menus reflected the sodium naturally found in foods. Similarly, the potassium content reflected the natural presence of potassium in foods as a salt substitute such as potassium chloride was not included in the menu planning and nutrient analysis. We presented the exact value of the phosphorus content of all diets that was reported in the database without factoring in the bioavailability of phosphorus sources. The phosphorus content may be underestimated because phosphorus-containing food additives were not accounted for in the food composition database.

We also calculated the Potential Renal Acid Load (PRAL) based on the nutrient composition of all types of LPDs and MHPDs using the formula as described by Remer et al. [24]:

PRAL (mEq/d) = 0.49 × protein (g/d) + 0.037 × phosphorus (mg/d) − 0.021 × potassium (mg/d) − 0.026 × magnesium (mg/d) − 0.013 × calcium (mg/d)

A diet with a positive PRAL value indicates acid is produced while a diet with a negative PRAL value indicates base is produced.

### 2.5. Defining Nutritional Reference Values 

We determined the adequacy of EAAs by referencing the Recommended Dietary Allowance (RDA) for adults (19 years and older) [25]. The recommendation for eicosapentaenoic acid (EPA) plus docosahexaenoic acid (DHA) was set at 250 mg/day [26]. The adequacy of dietary fiber was based on the Adequate Intake (AI) for total dietary fiber, which was 14 g/1000 kcal [25]. Since the energy prescription was 2100 kcal, the AI of the fiber was set at 29 g/day. We determined the adequacy of thiamine, riboflavin, niacin, folate, cobalamin, magnesium, copper, iron, and zinc according to their RDAs, while the AI for manganese was used as the reference value to determine adequacy [27,28,29]. CKD-specific recommendations were used as reference values for selected nutrients such as sodium, calcium, and pyridoxine [3,30]. There was no specific reference value for dietary phosphorus and potassium because the recommendation of the updated Kidney Disease Outcomes Quality Initiative Clinical Practice Guideline is to adjust the dietary intake of these two minerals to maintain serum phosphate and potassium within the normal range [3].

## 3. Results

The composition of macronutrients in the LPDs (0.5–0.8 g protein/kg/day) is presented in Table 2. The energy ranged from 2016–2229 kcal or 29–32 kcal/kg based on the reference body weight of 70 kg. The vegan LPDs had the greatest carbohydrate and fiber, whereas the lacto-ovo LPDs had the highest fat content. Only the conventional and PLADO LPDs met the WHO recommendations for EPA and DHA, while the EPA and DHA content in vegan and lacto-vegetarian LPDs was almost negligible.

**Figure 1:**
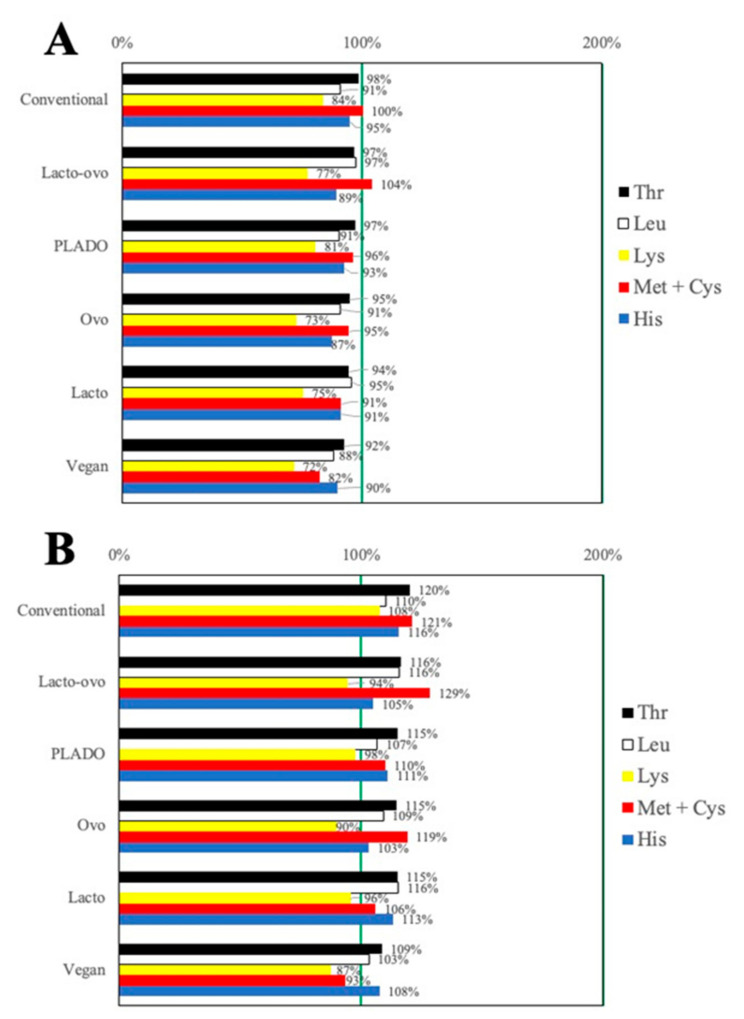
Percent of Recommended Dietary Allowance of selected essential amino acids for conventional, plant-based, and vegetarian diets at the protein prescription of (**A**) 0.5 g/kg/day and (**B**) 0.6 g/kg/day. Abbreviation: PLADO, plant-dominant. Amino acid abbreviations: Cys, cysteine; His, histidine; Leu, leucine; Lys, lysine; Met, methionine; Thr, threonine. Other essential amino acids such as tryptophan, isoleucine, phenylalanine, tyrosine, and valine are not included because all low protein diets meet the Recommended Dietary Allowance.

At the dietary protein prescription of 0.5 g/kg/day, no diet met the RDA for threonine, leucine, lysine, and histidine. Furthermore, the PLADO, lacto-vegetarian, ovo-vegetarian, and vegan diets did not meet the RDA for methionine + cysteine. The percent adequacy for these EAAs according to their RDA is presented in Figure 1A.

At the dietary protein prescription of 0.6 g/kg/day, the conventional diet met the RDA for all EAAs. The PLADO, ovo-vegetarian, lacto-vegetarian, and lacto-ovo-vegetarian diets were below the RDA only for lysine, whereas the vegan diet did not meet the RDAs for lysine and methionine + cysteine. The percent of adequacy for these EAAs according to the RDAs is presented in Figure 1B. With diets at 0.7 g protein/kg/day and greater, all plant-based and vegetarian diets as well as the conventional diet met the RDA for all EAAs.

The composition of macronutrients in the MHPDs (1.0–1.2 g/kg/day) is presented in Table 3. The energy ranged from 2079–2410 kcal or 20–34 kcal/kg based on the reference body weight of 70 kg. The vegan MHPDs had the greatest amount of carbohydrate and dietary fiber, whereas the lacto-ovo MHPDs had the highest fat content. Only the conventional and PLADO MHPDs met the WHO recommendations for EPA and DHA, whereas the EPA and DHA content in the vegan and lacto-vegetarian LPDs were almost negligible. All MHPDs met the RDA for each EAA.

The macromineral, trace element, and vitamin content of LPDs and MHPDs are presented in Table 4 and Table 5, respectively. The plant-based and vegetarian diets contained a greater amount of potassium, phosphorus, and calcium than the conventional diet. On the other hand, the conventional diet contained a relatively greater amount of sodium compared to the plant-based and vegetarian diets, although the sodium content was still below the amount usually recommended for CKD patients [3]. The plant-based and vegetarian LPDs, but not the conventional LPD, met the RDA for copper, whereas all MHPDs met the RDA for copper. All LPDs and MHPDs also met the AI for manganese. No LPD met the RDA for zinc, whereas only vegan MHPDs (≥1.0 g/kg/day), lacto-ovo-vegetarian MHPDs (≥1.1 g/kg/day), lacto-vegetarian MHPDs (≥1.1 g/kg/day), and ovo-vegetarian MHPDs (≥1.2 g/kg/day) were adequate. Similarly, no LPD met the RDA for magnesium, but vegan MHPDs (≥1.0 g/kg/day), lacto-vegetarian MHPDs (≥1.1 g/kg /day), and ovo-vegetarian MHPDs (≥1.2 g/kg /day) met the RDA for magnesium. All LPDs and MHPDs met the RDA for iron for both men and women above 50 years old (8 mg/day), whereas vegan diets (≥0.6 g/kg/day), ovo-vegetarian diets (≥0.7 g/kg/day), ovo-vegetarian diets (≥0.8 g/kg/day), PLADO diets (≥0.8 g/kg/day) and lacto-ovo-vegetarian diets (≥1.0 g/kg/day), but not the conventional diet, met the iron RDA for only women 19–50 years old (18 mg/day) [29].

For the B vitamins, all LPDs and MHPDs met the RDA for thiamine and folate, whereas no LPD or MHPD met the CKD-specific recommendation for pyridoxine. No LPD met the RDA for riboflavin except the lacto-ovo vegetarian LPDs (≥0.6 g/kg/day). The ovo-vegetarian and lacto-vegetarian diets at a protein prescription ≥1.1 g/kg/day met the RDA for riboflavin. None of the ovo-vegetarian and lacto-ovo-vegetarian diets met the RDA for niacin, whereas conventional LPDs (≥0.6 g/kg/day), PLADO LPDs (≥0.7 g/kg/day), vegan MHPDs (≥1.1 g/kg/day), and lacto-vegetarian MHPDs (≥1.2 g/kg/day) met the RDA for niacin. With regard to the RDA for cobalamin, none of the ovo-vegetarian, lacto-vegetarian, and vegan LPDs or MHPDs met this RDA, whereas the conventional diets (≥0.6 g/kg/day), PLADO diets (≥0.6 g/kg/day), and lacto-ovo-vegetarian diets (≥0.7 g/kg/day) met the RDA for cobalamin.

**Figure 2:**
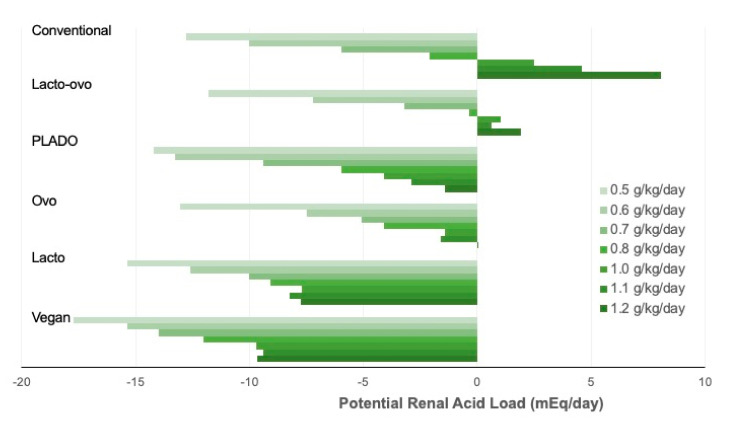
Potential renal acid load of all types of diets at different levels of protein prescription.

Figure 2 shows the PRAL of all types of LPDs and MHPDs. All PLADO, lacto-vegetarian, and vegan diets had negative PRAL, though at a decreasing trend when the protein prescription increased. The ovo-vegetarian diets had negative PRAL values at protein prescriptions from 0.5 to 1.1 g/kg body weight/day, and the PRAL value became positive at protein prescription of 1.2 g/kg body weight/day. The conventional and lacto-ovo-vegetarian diets had negative PRAL values at protein prescription below 0.8 g/kg body weight/day, and the PRAL values became positive at 1.0 g protein/kg body weight/day and above.

## 4. Discussion

The World Health Organization recommends that the safe level of protein intake for healthy adults is 0.83 g/kg body weight per day, for proteins with protein digestibility-corrected amino acid score value of 1.0 [31]. The LPDs (0.6–0.8 g/kg) prescribed to non-dialysis dependent CKD patients have been recommended to include at least 50% high biological value protein to ensure the adequacy of dietary needs [22]. Our LPD food pattern modeling concurred with this recommendation, since only the conventional LPD (0.6 g/kg/day) containing at least 50% of the animal-based protein met the RDA for all EAAs. However, the plant-based and vegetarian diets only met the RDA for all EAA with the protein prescription of >0.7 g/kg and above, and these EAAs are likely to be deficient when the protein intake is restricted to 0.6 g per kg body weight.

Plant proteins typically are less abundant than animal proteins in certain EAAs, especially methionine and lysine [15]. A cross-sectional study comparing the dietary intake and plasma concentration of amino acids among healthy men based on their habitual dietary intakes reported that vegans had the lowest dietary intake and plasma concentrations of lysine and methionine compared to meat-eaters, fish-eaters, and vegetarians [32]. Table 6 shows the content of selected EAA of proteins from different food sources in one exchange (7 g protein). For an LPD providing 0.6 g protein/kg/day or 42 g/day (using the reference body weight of 70 kg), three exchanges (21 g protein) of animal protein, such as fish or poultry, provide about 55–75% of the RDA for leucine, lysine, and methionine plus cysteine. However, when the animal proteins are substituted with plant protein such as *tofu*, lentils, or beans, the content of EAAs are expected to be lower, since three exchanges of these plant proteins provide only 50–55% of the RDA for leucine and lysine, and 30–40% of the RDA for methionine plus cysteine. As the protein prescription becomes higher (≥0.7 g/kg/day) and more plant proteins can be included in the diet, it is then possible for the diet to meet the RDAs for all EAAs.

Barsotti et al. [16] and Soraka et al. [18] demonstrated that a vegetarian LPD with a protein prescription of 0.70–0.75 g per kg body weight could meet their reported requirements for all EAAs. The authors noted that the patients prescribed with these diets did not experience a deterioration in nutritional status, even though the vegetarian LPD was proportionally lower in lysine, leucine, and methionine in comparison to both animal-based diets of lower protein content and the RDAs. There are several possible explanations for these observations. First, the RDA for a nutrient is determined if the dietary intake provides nutritionally sufficient amounts of that nutrient for about 97% of a normal population [25]. By this criterion, most people could ingest less than the RDA of a given nutrient and yet still receive a sufficient amount of that nutrient to be nutritionally adequate for them. Secondly, patients’ actual dietary protein intake might be more than what they were prescribed, at least some of the time [33]. Third, a limitation may arise from their nutritional assessment approach to determining patients’ protein-energy status which was not sufficiently sensitive as it relied only on body weight and serum proteins, whilst the patient’s vitamin, mineral, and trace element status were not evaluated by these studies.

To the best of our knowledge, the lowest protein content for a vegan diet for CKD patients that has been previously examined was 0.7 g/kg body weight/day, and the methods for assessing nutritional status in these patients were quite imprecise [16]. As shown in Table 6, plant proteins are inherently low in lysine and methionine. Therefore, achieving the RDA of these EAAs is very unlikely when the diet is low in protein (0.6 g/kg) and contains exclusively plant proteins at the same time. The PLADO low protein diet (0.6 g/kg) consisting of only 30% animal proteins was also deficient in lysine. In fact, our data indicate that the conventional low protein diet (0.6 g/kg) with 50% animal proteins also marginally met the RDA for lysine (108%). Nonetheless, many nitrogen balance studies indicate that a 0.60 g protein/kg/day diet providing about 50% of protein of high biological value (i.e., animal protein) will maintain neutral or positive nitrogen balance in advanced CKD patients who are not undergoing dialysis therapy [22,34].

Although the data from this food pattern modeling indicated that a well-planned plant-based or vegetarian LPD (≥0.7 g/kg/day) can meet the RDA for all EAAs, the RDA is not CKD-specific. It is possible that there are alterations in the dietary requirements for some EAAs in people with CKD, especially if such individuals have comorbid conditions [35,36,37,38]. Patients with advanced CKD have altered plasma and tissue amino acid profiles [39,40,41]. There is evidence for intracellular taurine depletion in nondialyzed CKD and maintenance hemodialysis patients [35,36,42], and plant foods are markedly deficient in taurine [43]. Therefore, a plant-based or a vegan diet may be unable to supply a sufficient amount of taurine or other sulfur containing amino acids to maintain normal cellular taurine levels [44], and CKD and chronic dialysis patients consuming vegan diets could be at risk for taurine deficiency. Future studies are indicated to confirm that these plant-based and especially vegan LPDs are nutritionally adequate for CKD patients. These considerations also suggest that CKD and chronic dialysis patients adhering to a vegan LPD should be monitored especially closely to assess their nutritional status and to prevent protein-energy wasting. This issue may be especially challenging in low- and middle-income Asian countries with limited dietitian accessibility [45] and where malnutrition risk may be particularly high, chronic infections such as tuberculosis are endemic [46], and populations have marginal protein intakes [47].

A recent review of observation studies by Picard et al. [48] reported that free-living CKD patients consuming vegetarian or plant-based diets had a significantly lower self-reported dietary protein intake. However, the effects of this lower protein intake on the patients’ protein-energy status were not carefully assessed; only serum albumin levels were reported, and serum albumin, which was not different in the patients consuming the animal-based vs. the plant-based diets, is rather a more sensitive indicator of inflammation than nutritional status [49]. Nevertheless, this review supports the contention that CKD patients prescribed LPDs, and especially plant-based or vegetarian LPDs, should be counselled by experienced dietitians, and their protein-energy status should be periodically monitored [48]. An epidemiological study of Chinese maintenance hemodialysis patients suggested that the proportion of plant-based proteins in their diet bore a U-curve relationship with adjusted all-cause mortality and cardiovascular mortality [49]. The lowest adjusted mortality rates were observed in the maintenance dialysis patients who ingested plant-based and animal-based proteins in approximately a 0.45 ratio. Hence, 55 percent of the food intake was animal-based for this data. This study was limited by the fact that the assessment of food intake was made for three separate 24 h periods at the beginning of the median follow-up period of 28 months [50].

The preceding discussion indicates that plant-based LPDs may place CKD patients at increased risk for nutritional deficiencies, especially if these diets provide less than 0.7 g protein/kg/day. However, plant-based diets might also increase the intake of other nutrients to unwanted levels. For CKD patients, this may be particularly the case for potassium and phosphorus, which could exacerbate electrolyte imbalances in patients with CKD [10]. Indeed, the vegetarian LPDs and MHPDs contained greater amounts of potassium and phosphorus than the conventional and plant-based diets, as shown in Table 4 and Table 5. This is especially true for the vegan diets. The sodium content of these diets reflected only the sodium found naturally in foods, and the amount was substantially lower than what a person would normally consume. As condiments and seasonings were not included in nutrient analysis, the potassium and phosphorus contents were also underestimated because certain condiments commonly used in Chinese cuisine such as soy sauce, oyster sauce, and fish sauce also contain significant amounts of potassium and phosphorus [51].

In this study, we did not include vegetarian meat substitutes that may contain substantial amounts of sodium, potassium, and phosphorus [52]. In addition, these plant-based meat alternatives are low in certain nutrients such as zinc, calcium, and vitamin B12 [53]. Therefore, natural or unprocessed plant-based proteins were chosen. Although the phosphorus of plant-based foods is generally believed to have lower bioavailability (10–30%) due to the phytates in these diets, a review of human intervention studies suggests that a much greater amount of phytate-phosphorus is absorbed (at least 50%). Commercial food processing also increases the amount of bioavailable phosphorus. Therefore, the phosphorus of plant proteins, particularly those that are highly processed, should not be assumed to be poorly absorbed [54]. Nevertheless, the phosphorus bioavailability of plant foods is still lower than animal foods. On the other hand, the dairy products included in the lacto- and lacto-ovo vegetarian diets are significant sources of dietary phosphorus and calcium. The high dietary calcium content might increase the risk of calcium deposition in tissues, which is a not an uncommon occurrence amongst advanced CKD patients [55].

Plant-based and vegetarian diets may also lack some other essential nutrients that are more prevalent in foods of animal origin, such as long-chain *n*-3 PUFAs and vitamin B-12 or cobalamin [56]. A cross-sectional study showed that fish-eaters had a greater dietary intake of long-chain *n*-3 PUFAs than vegetarians or vegans. However, the difference in plasma long-chain *n*-3 PUFAs status between fish-eaters and vegetarians or vegans was smaller than expected, possibly due to a greater estimated precursor-product ratio of dietary α-linolenic acid to long-chain *n*-3 PUFAs among vegetarians or vegans [57]. Therefore, patients adhering to a plant-based or vegetarian diet should be encouraged to consume foods high in α-linolenic acid to ensure optimal plasma *n*-3 PUFAs status. On the other hand, since vitamin B-12 is obtained exclusively from animal sources, CKD patients adhering to a vegetarian diet must consume vitamin B-12 fortified foods or dietary supplements to prevent deficiency [5].

On the other hand, it is worthwhile to highlight that the planned plant-based and vegetarian diets contained a relatively greater amount of dietary fiber than the conventional diet that was basically animal-based. Meta-analyses of experimental studies have demonstrated the benefits of dietary fiber supplements (dosage ranging from 7 to 50 g/day) in reducing serum urea, creatinine, and *p*-cresyl sulphate [58,59]. In our study, the fiber content of the plant-based or vegetarian LPDs (0.5–0.8 g/kg/day) was greater than the conventional LPDs by 8–20 g/day. In addition, plant proteins such as beans, legumes, and lentils have a lower PRAL than animal proteins such as meat, cheese, and eggs [60]. Both epidemiological studies [61,62] and clinical trials [63,64] have indicated that even mild acidosis can be associated with more rapid progression of CKD, and a diet that is low in its PRAL may slow such progression [65,66]. However, our study showed that the PRAL values of the conventional LPDs were not substantially higher than the plant-based or vegetarian diets when the protein content was low (0.5–0.6 g/kg body weight/day).

Plant proteins also do not contain cholesterol and have a lower saturated fat content than animal proteins. However, the evidence does not indicate that advanced CKD or chronic dialysis patients who ingest lower cholesterol diets or who are taking statins have less cardiovascular disease or reduced mortality [67]. Consumption of plant-based foods is proposed to confer planetary benefits as negative impacts of large-scale meat production on the environment in terms of the land use, water use, and the emission of greenhouse gases have been highlighted [68]. It should be considered though that for many Asian countries, animal proteins are often derived from seafood, poultry or pork, rather than from cattle [69]. With caution, plant proteins may be encouraged for individuals with CKD as long as the diet is carefully planned to ensure that the overall nutritional adequacy is met. As our study suggests, the 0.60 g protein/kg/day diet, it may be difficult to ensure sufficient amounts of all of the EAAs without including animal protein.

Our study had some limitations. Firstly, the LPD and MHPD modeling was based on an ideal menu planning that is theoretical in nature and therefore may not necessarily reflect habitual dietary intakes of any individual CKD patient. Secondly, the modeling study was based on food choices commonly consumed in Asian cuisine, mainly Chinese and Indian. Therefore, these findings may not be generalized to other Asian food cultures, but analysis of these Chinese and Indian hospital-based diets may provide useful insights to food choices across the Asian spectrum. In addition, we determined the amino acid content using a food composition database (FoodData Central) rather than by direct laboratory analytical procedures. In addition, variability in the composition of foods may exist between regions and between countries. However, this concern also raises the possibility that some foodstuffs might contain fewer essential nutrients than is reported in this database. Moreover, we did not include protein-free products (e.g., pasta, bread, and flour) in planning the LPDs, which, if included, would reduce the proportion of protein from cereals and allow the inclusion of a greater amount of protein food sources for the LPDs. However, such products are atypical to Asian societies and therefore have limited availability in food retail, whereas rice is the staple cereal of choice in the Asian region [70]. Lastly, the actual phosphorus content of the diets may be substantially higher than is indicated in the food composition database because the widely prevalent use of phosphorus-based additives was not taken into account and potentially available in ready-to-use spice powders, flavorings, and ultraprocessed foods available to Indian and Chinese societies. Moreover, the varying bioavailability of phosphorus in different food sources was not addressed in our calculations.

## 5. Conclusions

Our food pattern modeling indicated that the Asian plant-based and vegetarian diets providing 0.7 g protein/kg/day or more could meet the RDA for all EAAs. However, at the protein prescription of 0.6 g/kg/day, only the conventional diet consisting of 50% high biological protein from animal-based foods is able to meet the RDA of all EAAs, while the vegetarian or plant-based LPDs are likely to be deficient in EAAs. Therefore, a higher protein level (at least 0.7 g protein/kg/day) should be considered for individuals who wish to adhere to vegetarian or plant-based diets, and these diets must be carefully planned because the protein prescription is still below the RDA. With regards to macrominerals, plant-based and vegetarian diets contained a considerable amount of potassium, phosphorus, and calcium as compared to the conventional diet. The conventional diet was low in essential nutrients such as zinc, copper, magnesium, riboflavin, and pyridoxine while plant-based and vegetarian diets were low in *n*-3 long-chain polyunsaturated fatty acids, magnesium, riboflavin, niacin, pyridoxine, and cobalamin. The use of a multivitamin and trace element supplement should be considered with these diets. The foregoing data indicate that prescription of all LPDs for CKD patients requires careful planning and continued monitoring.

## Figures and Tables

**Table 1:** Macronutrient distribution and food groups of a conventional diet.

	LPD (g/kg BW)				MHPD (g/kg BW)		
	0.5	0.6	0.7	0.8	1.0	1.1	1.2
Nutrients *							
Energy (kcal)	2100	2100	2100	2100	2100	2100	2100
Protein (% EN)	7	8	9	11	13	15	16
Protein (g)	35	42	49	56	70	77	84
Carbohydrate (% EN)	58	57	56	54	54	54	54
Carbohydrate (g)	305	299	294	284	284	284	284
Fat (% EN)	35	35	35	35	33	31	30
Fat (g)	82	82	82	82	77	72	70
**Food groups ^†^**							
Cereals (exchange)	8	8	8	8	13	13	13
Fruits (exchange)	3	3	3	3	3	3	3
Non-starchy vegetables (serving)	4	4	4	4	4	4	4
Poultry/fish (exchange)	2	3	4	4	5	6	6
Egg (exchange)	0	0	0	1	1	1	2
Sugar (exchange)	5	4	4	3	0	0	0
Fat (exchange)	14	14	14	14	13	12	11

Abbreviations: BW, body weight; EN, energy; LPD, low protein diet; MHPD, moderately high protein diet. * Based on a body weight of 70 kg. ^†^ One exchange of cereal contains 15 g carbohydrate, 2 g protein, 0.5 g fat; one exchange of fruit or sugar contains 15 g carbohydrate; one exchange of poultry, fish, or egg contains 7 g protein; one exchange of fat contains 5 g fat; the portion size of one serving of cooked non-starchy vegetable is ½ cup.

**Table 2:** Macronutrients, essential amino acid, and long-chain *n*-3 polyunsaturated fatty acid content of conventional, plant-based, and vegetarian low protein diets.

Nutrients	Reference ^†^	0.5 g/kg/Day or 35 g/Day *						0.6 g/kg/Day or 42 g/Day *					
		Conventional	Lacto-ovo	PLADO	Ovo	Lacto	Vegan	Conventional	Lacto-ovo	PLADO	Ovo	Lacto	Vegan
Energy (kcal)		2016	2071	2047	2078	2090	2098	2059	2145	2088	2100	2140	2138
Carbohydrate (g)		300	301	308	311	312	324	300	302	315	298	306	325
Dietary fiber (g)	29 ^a^	19	19	21	23	23	27	19	19	23	23	24	32
Total protein (g)		36	36	36	36	36	36	43	42	42	42	43	42
Animal protein (g)		15	13	11	6	8	0	22	19	12	13	11	0
Plant protein (g)		21	23	25	30	28	36	21	23	30	29	32	42
Essential AA													
Tryptophan (mg)	350 ^a^	409	441	409	417	442	408	487	524	488	500	540	470
Threonine (mg)	1400 ^a^	1377	1353	1359	1326	1323	1293	1681	1631	1611	1604	1610	1520
Isoleucine (mg)	1330 ^a^	1608	1653	1586	1579	1613	1531	1927	1989	1879	1915	1948	1802
Leucine (mg)	2940 ^a^	2678	2866	2663	2667	2807	2589	3240	3410	3136	3211	3396	3035
Lysine (mg)	2660 ^a^	2226	2056	2147	1934	2006	1906	2861	2513	2601	2391	2544	2326
Methionine + Cysteine (mg)	1330 ^a^	1333	1384	1279	1257	1213	1095	1612	1710	1462	1583	1411	1243
Phenylalanine + Tyrosine (mg)	2310 ^a^	2715	3022	2738	2867	2953	2779	3218	3612	3234	3457	3561	3258
Valine (mg)	1680 ^a^	1895	2075	1872	1962	1979	1833	2251	2505	2171	2391	2346	2121
Histidine (mg)	980 ^a^	930	875	908	857	895	881	1134	1029	1089	1011	1108	1054
Total fat (g)		77	83	77	80	80	78	78	88	77	85	86	79
EPA+DHA (mg)	250 ^b^	263	21	260	21	0	0	537	42	279	42	0	0
**Nutrients**	**Reference ^†^**	0.7 g/kg/Day or 49 g/Day *						0.8 g/kg/Day or 56 g/Day *					
		**Conventional**	**Lacto-ovo**	**PLADO**	**Ovo**	**Lacto**	**Vegan**	**Conventional**	**Lacto-ovo**	**PLADO**	**Ovo**	**Lacto**	**Vegan**
Energy (kcal)		2077	2170	2180	2146	2185	2181	2083	2195	2143	2189	2229	2227
Carbohydrate (g)		295	294	318	299	309	325	282	287	299	300	311	327
Dietary fiber (g)	29 ^a^	19	20	27	28	28	36	19	25	30	32	31	40
Total protein (g)		50	50	50	49	50	49	56	56	57	55	55	55
Animal protein (g)		29	25	15	13	14	0	35	25	17	13	14	0
Plant protein (g)		21	25	35	36	36	49	21	31	40	42	41	55
Essential AA													
Tryptophan (mg)	350 ^a^	567	623	572	574	606	545	641	689	638	649	671	619
Threonine (mg)	1400 ^a^	1970	1919	1923	1863	1846	1765	2243	2160	2164	2108	2056	2024
Isoleucine (mg)	1330 ^a^	2288	2374	2250	2223	2276	2090	2582	2659	2526	2511	2524	2399
Leucine (mg)	2940 ^a^	3753	4119	3718	3715	3970	3501	4239	4590	4182	4181	4368	4005
Lysine (mg)	2660 ^a^	3443	3112	3180	2862	3036	2756	3989	3550	3652	3291	3399	3226
Methionine + Cysteine (mg)	1330 ^a^	1889	2008	1714	1753	1664	1407	2132	2166	1894	1916	1806	1576
Phenylalanine + Tyrosine (mg)	2310 ^a^	3721	4315	3849	3999	4143	3761	4175	4821	4311	4502	4574	4302
Valine (mg)	1680 ^a^	2590	2948	2530	2718	2719	2421	2913	3252	2839	3018	2972	2748
Histidine (mg)	980 ^a^	1346	1245	1318	1206	1288	1233	1567	1425	1493	1385	1441	1428
Total fat (g)		79	91	82	87	87	81	83	93	83	89	89	83
EPA+DHA (mg)	250 ^b^	543	42	282	42	0	0	603	42	400	42	0	0

Abbreviations: AA, amino acid, DHA, docosahexaenoic acid; EPA, eicosapentaenoic acid; PLADO, plant-dominant. Note: Table cells with grey shading indicate values below the reference value. * Represents dietary nutrient content for a 70 kg person. ^†^ The Reference values refer to Adequate Intake for dietary fiber [25], Recommended Daily Allowances for essential amino acids [25], and the recommended dietary requirement by FAO/WHO for *n*-3 polyunsaturated fatty acids [26]. ^a^ Dietary Reference Intakes for Energy, Carbohydrate, Fiber, Fat, Fatty Acids, Cholesterol, Protein, and Amino Acids (2005) [25]. ^b^ Joint FAO/WHO Expert Consultation on Fats and fatty acids in Human Nutrition (2008) [26].

**Table 3:** Macronutrients, essential amino acids, and long-chain *n*-3 polyunsaturated fatty acids of conventional, plant-based, and vegetarian moderately high protein diets.

Nutrients	Reference ^†^	1.0 g/kg/Day or 70 g/Day *						1.1 g/kg/Day or 77 g/Day *						1.2 g/kg/Day or 84 g/Day *					
		Conventional	Lacto-ovo	PLADO	Ovo	Lacto	Vegan	Conventional	Lacto-ovo	PLADO	Ovo	Lacto	Vegan	Conventional	Lacto-ovo	PLADO	Ovo	Lacto	Vegan
Energy (kcal)		2140	2220	2096	2273	2224	2295	2097	2265	2142	2247	2306	2353	2079	2290	2201	2298	2340	2410
Carbohydrate (g)		286	292	289	302	315	335	286	300	297	308	331	349	286	313	310	324	345	366
Dietary fiber (g)	29 ^a^	23	32	34	40	41	48	25	34	38	42	45	54	25	38	42	46	49	58
Total protein (g)		70	71	70	69	69	70	77	78	79	76	77	78	84	85	86	83	85	84
Animal protein (g)		44	25	23	13	13	0	50	25	25	15	14	0	58	25	28	15	14	0
Plant protein (g)		26	46	47	56	56	70	27	53	54	61	63	78	26	60	58	68	71	84
Essential AA																			
Tryptophan (mg)	350 ^a^	801	865	792	800	818	790	874	976	896	891	925	869	959	1048	963	962	1012	936
Threonine (mg)	1400 ^a^	2823	2687	2660	2599	2547	2562	3095	3001	3020	2886	2864	2837	3417	3232	3279	3128	3140	3067
Isoleucine (mg)	1330 ^a^	3228	3314	3113	3073	3137	3011	3588	3714	3529	3443	3512	3333	3960	3983	3818	3726	3839	3609
Leucine (mg)	2940 ^a^	5325	5711	5085	5127	5449	5024	5864	6339	5776	5705	6041	5551	6447	6786	6267	6166	6565	6002
Lysine (mg)	2660 ^a^	5062	4520	4461	4053	4385	4043	5636	5023	5140	4549	4891	4538	6302	5433	5644	4974	5370	4962
Methionine +	1330 ^a^	2626	2560	2244	2204	2166	1905	2933	2849	2497	2476	2374	2085	3237	3005	2698	2645	2557	2228
Cysteine (mg)																			
Phenylalanine +	2310 ^a^	5279	5983	5268	5552	5672	5441	5796	6671	5969	6182	6316	6006	6340	7154	6463	6687	6882	6483
Tyrosine (mg)																			
Valine (mg)	1680 ^a^	3631	3943	3468	3648	3645	3408	4022	4400	3930	4065	4040	3753	4400	4684	4241	4346	4379	4061
Histidine (mg)	980 ^a^	1999	1838	1833	1731	1850	1801	2188	2046	2095	1930	2065	2004	2415	2220	2280	2108	2267	2179
Total fat (g)		81	89	77	92	81	81	73	87	75	83	80	78	67	82	73	79	75	74
EPA+DHA (mg)	250 ^b^	743	42	406	42	0	0	805	42	523	42	0	0	906	42	561	42	0	0

Abbreviations: AA, amino acid, DHA, docosahexaenoic acid; EPA, eicosapentaenoic acid; PLADO, plant-dominant. Note: Table cells with grey shading indicate values below the reference value. * Represents dietary nutrient content for a 70 kg person. ^†^ The Reference values refer to Adequate Intake for dietary fiber [25], Recommended Daily Allowances for essential amino acids [25], and the recommended dietary requirement by FAO/WHO for *n*-3 polyunsaturated fatty acids [26]. ^a^ Dietary Reference Intakes for Energy, Carbohydrate, Fiber, Fat, Fatty Acids, Cholesterol, Protein, and Amino Acids (2005) [25]. ^b^ Joint FAO/WHO Expert Consultation on Fats and fatty acids in Human Nutrition (2008) [26].

**Table 4:** Macromineral, trace element, and vitamin content of conventional, plant-based, and vegetarian low protein diets.

Nutrients	Reference ^‡^		0.5 g/kg/Day or 35 g/Day ^†^						0.6 g/kg/Day or 42 g/Day ^†^					
	Male	Female	Conven-tional	Lacto-ovo	PLADO	Ovo	Lacto	Vegan	Conven-tional	Lacto-ovo	PLADO	Ovo	Lacto	Vegan
Sodium * (mg)	<2300 ^a^		1088	573	1082	611	692	548	1120	643	758	681	728	551
Potassium (mg)			1936	1952	2003	1992	2114	2149	2093	2019	2135	2009	2212	2311
Phosphorus (mg)			525	670	563	632	705	646	606	766	651	725	818	759
P/Prot ratio			14.5	18.6	15.5	17.7	19.4	18.0	14.1	18.1	15.4	17.2	19.0	18.0
Calcium (mg)	800–1000 ^a^		264	557	341	457	634	551	270	584	486	484	812	604
Magnesium (mg)	420 ^b^	320 ^b^	222	230	237	243	248	269	233	236	260	247	266	294
Copper (μg)	900 ^c^		838	930	965	1101	1016	1256	854	981	1122	1133	1100	1434
Manganese (mg)	2.3 ^c^	1.8 ^c^	6.1	6.1	6.5	6.7	6.4	7.3	6.1	6.1	6.9	6.2	6.2	7.2
Iron (mg)	8 ^c^	18 ^c,d^	11	13	13	15	14	17	11	14	15	16	16	20
Zinc (mg)	11 ^c^	8 ^c^	5	6	5	6	6	6	5	6	6	6	7	7
Thiamine (mg)	1.2 ^e^	1.1 ^e^	1.2	1.2	1.2	1.2	1.3	1.3	1.2	1.2	1.2	1.3	1.3	1.4
Riboflavin (mg)	1.3 ^e^	1.1 ^e^	0.7	1.1	0.7	0.9	1.0	0.7	0.8	1.3	0.8	1.1	1.1	0.7
Niacin (mg)	16 ^e^	14 ^e^	15	11	14	11	11	12	17	11	15	11	11	12
Pyridoxine (mg)	5.0 ^f^		1.3	1.1	1.3	1.2	1.1	1.2	1.4	1.2	1.3	1.3	1.2	1.3
Folate (μg)	400 ^e^		483	529	532	613	584	674	484	552	584	624	604	770
Cobalamin (μg)	2.4 ^e^		2.3	1.3	2.2	0.6	0.8	0.0	5.3	1.9	3.1	1.3	1.1	0.0
**Nutrients**	**Reference ^‡^**		**0.7 g/kg/Day or 49 g/Day** **^†^**						**0.8 g/kg/Day or 56 g/Day** **^†^**					
	**Male**	**Female**	**Conven-tional**	**Lacto-ovo**	**PLADO**	**Ovo**	**Lacto**	**Vegan**	**Conven-tional**	**Lacto-ovo**	**PLADO**	**Ovo**	**Lacto**	**Vegan**
Sodium * (mg)	<2300 ^a^		1135	626	1106	685	492	558	1151	900	1114	692	497	561
Potassium (mg)			2121	2163	2282	2183	2458	2486	2157	2308	2406	2357	2585	2660
Phosphorus (mg)			644	936	765	846	981	866	694	1054	861	954	1069	988
P/Prot ratio			12.9	18.8	15.2	17.3	19.6	17.9	12.4	18.7	15.2	17.2	19.3	17.8
Calcium (mg)	800–1000 ^a^		273	796	517	569	891	724	277	851	561	689	1006	809
Magnesium (mg)	420 ^b^	320 ^b^	238	252	290	275	292	324	242	279	305	304	316	352
Copper (μg)	900 ^c^		857	1012	1313	1329	1241	1621	850	1188	1395	1516	1404	1817
Manganese (mg)	2.3 ^c^	1.8 ^c^	5.9	5.5	7.0	6.3	6.0	7.3	5.4	5.2	6.2	6.3	6.1	7.3
Iron (mg)	8 ^c^	18 ^c,d^	12	14	17	19	17	23	12	17	19	22	20	26
Zinc (mg)	11 ^c^	8 ^c^	5	7	7	7	8	8	6	8	7	8	9	9
Thiamine (mg)	1.2 ^e^	1.1 ^e^	1.2	1.3	1.3	1.4	1.4	1.5	1.3	1.4	1.4	1.5	1.5	1.6
Riboflavin (mg)	1.3 ^e^	1.1 ^e^	0.8	1.5	0.8	1.1	1.2	0.7	0.8	1.5	0.8	1.1	1.2	0.8
Niacin (mg)	16 ^e^	14 ^e^	19	11	16	12	12	13	20	11	17	12	12	13
Pyridoxine (mg)	5.0 ^f^		1.5	1.2	1.5	1.4	1.3	1.4	1.6	1.3	1.6	1.5	1.4	1.5
Folate (μg)	400 ^e^		481	566	668	722	700	843	470	655	708	795	759	941
Cobalamin (μg)	2.4 ^e^		5.3	2.5	3.1	1.3	1.4	0.0	5.8	2.5	3.8	1.3	1.4	0.0

Abbreviations: P/Prot, phosphorus/protein; PLADO, plant-dominant. Note: Table cells with grey shading indicate values below or exceeding the reference value. * Only sodium naturally found in food without condiments added during cooking, table salt, or sauces. ^†^ Represents dietary nutrient content for a 70 kg person. **^‡^** The reference value refers to the CKD-specific recommendation for dietary sodium, calcium [3], and pyridoxine [30]; Recommended Dietary Allowance for magnesium, copper, iron, zinc, thiamine, riboflavin, niacin, folate, and cobalamin [27,28,29] and Adequate Intake for manganese [29]. ^a^ KDOQI Clinical Practice Guideline for Nutrition in CKD: 2020 Update [3]. ^b^ Dietary Reference Intakes for Calcium, Phosphorus, Magnesium, Vitamin D, and Fluoride (1997) [27]. ^c^ Dietary Reference Intakes for Vitamin A, Vitamin K, Arsenic, Boron, Chromium, Copper, Iodine, Iron, Manganese, Molybdenum, Nickel, Silicon, Vanadium, and Zinc (2000) [29]. ^d^ RDA for women 19–50 years old only. The RDA for women >50 years old is 8 mg/day [29]. ^e^ Dietary Reference Intakes for Thiamin, Riboflavin, Niacin, Vitamin B6, Folate, Vitamin B12, Pantothenic Acid, Biotin, and Choline (1998) [28]. ^f^ Kopple et al. 1981 [30].

**Table 5:** Macromineral, trace element, and vitamin content of standard, plant-based, and vegetarian moderately high protein diets.

Nutrients	Reference ^‡^		1.0 g/kg/Day or 70 g/Day ^†^						1.1 g/kg/Day or 77 g/Day ^†^						1.2 g/kg/Day or 84 g/Day ^†^					
	Male	Female	Conven-tional	Lacto-ovo	PLADO	Ovo	Lacto	Vegan	Conven-tional	Lacto-ovo	PLADO	Ovo	Lacto	Vegan	Conventional	Lacto-ovo	PLADO	Ovo	Lacto	Vegan
Sodium * (mg)	<2300 ^a^		1059	691	882	786	559	658	1107	749	894	828	583	667	969	755	918	834	590	672
Potassium (mg)			2471	2670	2701	2745	2988	3045	2602	2909	3003	2937	3317	3351	2698	3087	3245	3119	3552	3623
Phosphorus (mg)			854	1258	981	1160	1281	1199	899	1363	1129	1237	1434	1336	961	1467	1242	1347	1554	1452
P/Prot ratio			12.2	17.8	14.1	16.8	18.5	17.1	11.7	17.4	14.3	16.2	18.5	17.2	11.4	17.4	14.5	16.3	18.3	17.3
Calcium (mg)	800–1000 ^a^		320	1173	682	830	1241	1023	326	1328	796	971	1413	1118	330	1442	821	1064	1567	1198
Magnesium (mg)	420 ^b^	320 ^b^	283	346	350	373	385	428	289	372	384	397	423	467	296	401	411	429	458	496
Copper (μg)	900 ^c^		997	1551	1536	1867	1769	2224	988	1670	1728	2000	1988	2460	994	1867	1882	2229	2212	2640
Manganese (mg)	2.3 ^c^	1.8 ^c^	5.3	5.5	5.6	6.1	6.1	7.5	5.1	5.8	5.9	6.5	6.7	8.0	5.1	6.3	6.2	7.1	7.3	8.5
Iron (mg)	8 ^c^	18 ^c,d^	14	23	22	27	26	31	14	25	25	29	29	34	14	28	27	32	33	37
Zinc (mg)	11 ^c^	8 ^c^	7	10	8	10	10	11	7	11	9	10	11	12	7	11	10	11	12	13
Thiamine (mg)	1.2 ^e^	1.1 ^e^	1.6	1.6	1.6	1.8	1.8	1.9	1.6	1.7	1.8	1.8	1.9	2.0	1.6	1.8	1.9	1.9	2.0	2.1
Riboflavin (mg)	1.3 ^e^	1.1 ^e^	1.0	1.6	0.9	1.2	1.2	0.8	1.1	1.8	0.9	1.3	1.4	0.9	1.2	1.9	1.0	1.4	1.4	1.0
Niacin (mg)	16 ^e^	14 ^e^	24	13	20	14	14	15	24	14	21	15	15	16	27	14	23	15	16	17
Pyridoxine (mg)	5.0 ^f^		1.8	1.5	1.8	1.7	1.6	1.8	1.9	1.6	2.0	1.8	1.8	2.0	2.0	1.7	2.2	1.9	1.9	2.1
Folate (μg)	400 ^e^		537	797	746	925	915	1065	554	838	840	965	1028	1178	554	897	922	1062	1114	1279
Cobalamin (μg)	2.4 ^e^		7.3	2.5	4.8	2.3	1.2	1.0	7.7	2.7	5.4	2.3	1.4	1.0	9.8	2.7	7.1	2.3	1.4	1.0

Abbreviations: P/Prot, phosphorus/protein; PLADO, plant-dominant. Note: Table cells with grey shading indicate values below or exceeding the reference value. * Only sodium naturally found in food without condiments added during cooking, table salt, or sauces. ^†^ Represents dietary nutrient content for a 70 kg person. **^‡^** The reference value refers to the CKD-specific recommendation for dietary sodium, calcium [3], and pyridoxine [30]; Recommended Dietary Allowance for magnesium, copper, iron, zinc, thiamine, riboflavin, niacin, folate, and cobalamin [27,28,29] and Adequate Intake for manganese [29]. ^a^ KDOQI Clinical Practice Guideline for Nutrition in CKD: 2020 Update [3]. ^b^ Dietary Reference Intakes for Calcium, Phosphorus, Magnesium, Vitamin D, and Fluoride (1997) [27]. ^c^ Dietary Reference Intakes for Vitamin A, Vitamin K, Arsenic, Boron, Chromium, Copper, Iodine, Iron, Manganese, Molybdenum, Nickel, Silicon, Vanadium, and Zinc (2000) [29]. ^d^ RDA for women 19–50 years old only. The RDA for women >50 years old is 8 mg/day [28]. ^e^ Dietary Reference Intakes for Thiamin, Riboflavin, Niacin, Vitamin B6, Folate, Vitamin B12, Pantothenic Acid, Biotin, and Choline (1998) [28]. ^f^ Kopple et al. 1981 [30].

**Table 6:** Selected essential amino acids of protein food sources (7 g protein).

	Fish (King Mackerel)	Chicken	Egg	Whole Milk	Tofu	Lentils	Chickpeas
Weight (g)	35	30	56	220	87	78	80
Protein (g)	7	7	7	7	7	7	7
Leucine (mg)	577	520	609	583	534	510	505
Leucine RDA * (%)	20	18	21	20	18	17	17
Lysine (mg)	652	589	512	308	463	491	474
Lysine RDA * (%)	25	22	19	12	17	18	18
Methionine (mg)	210	192	213	165	90	60	93
Cysteine (mg)	76	89	152	37	97	92	95
Methionine + cysteine RDA * (%)	22	21	27	15	14	11	14

Source: FoodData Central Database of the United States Department of Agriculture [23]. * The RDAs for Leucine, Lysine, and Methionine + Cysteine were 2940 mg, 2660 mg, and 1330 mg (based on the reference body weight of 70 kg).

## Data Availability

No new data were created in this study. Data sharing is not applicable to this article.

## Supplementary Materials

The following are available online at https://www.mdpi.com/article/10.3390/nu13103341/s1, Table S1: Three days sample menu.
