# Supplementary material for: Nutritional Adequacy of Animal-Based and Plant-Based Asian Diets for Chronic Kidney Disease Patients: A Modeling Study
Source: Nutrients. 2021 Sep 24;13(10):3341. doi: 10.3390/nu13103341 (PMC8541170; doi:10.3390/nu13103341)
Supplement: Supplementary file 1 [file nutrients-13-03341-s001.zip › nutrients-1382245-supplementary.pdf]

Sample Menu for Low Protein Diet (LPD) – 2100 kcal, 0.5 g/kg/d or 35 g/d protein

DAY 1 (South Indian Menu)

Conventional	Vegan	Ovo-vegetarian	Lacto-vegetarian	Lacto-ovo vegetarian	Plant-dominant
**Breakfast**<br>2 pc dosa<br>2 tbsp curry gravy<br>1 cup tea, 1 tbsp sugar	**Breakfast**<br>2 pc dosa<br>2 tbsp curry gravy<br>1 cup tea, 1 tbsp sugar	**Breakfast**<br>2 pc dosa<br>2 tbsp curry gravy<br>1 cup tea, 1 tbsp sugar	**Breakfast**<br>2 pc dosa<br>2 tbsp curry gravy<br>1 cup tea, 1 tbsp sugar	**Breakfast**<br>2 pc dosa<br>2 tbsp curry gravy<br>1 cup tea, 1 tbsp sugar	**Breakfast**<br>2 pc dosa<br>2 tbsp curry gravy<br>1 cup tea, 1 tbsp sugar
**Morning snack**<br>½ pc mango<br>1 cup tea, 1 tbsp sugar	**Morning snack**<br>½ pc mango<br>1 cup tea, 1 tbsp sugar	**Morning snack**<br>½ pc mango<br>1 cup tea, 1 tbsp sugar	**Morning snack**<br>½ pc mango<br>1 cup tea, 1 tbsp sugar	**Morning snack**<br>½ pc mango<br>1 cup tea, 1 tbsp sugar	**Morning snack**<br>½ pc mango<br>1 cup tea, 1 tbsp sugar
**Lunch**<br>1 medium bowl white rice<br>¼ cup okra in tamarind gravy<br>¼ cup French beans & carrot<br>Chicken curry:<br>1 oz chicken<br>2 tbsp curry gravy<br>1 small bowl rasam<br>1 cup tea, 1 tbsp sugar	**Lunch**<br>1 medium bowl white rice<br>¼ cup okra in tamarind gravy<br>¼ cup French beans & carrot<br>Chickpeas curry:<br>½ cup chickpea<br>2 tbsp curry gravy<br>1 small bowl rasam<br>1 cup tea, 1 tbsp sugar	**Lunch**<br>1 medium bowl white rice<br>¼ cup okra in tamarind gravy<br>¼ cup French beans & carrot<br>Chickpeas curry:<br>½ cup chickpea<br>2 tbsp curry gravy<br>1 small bowl rasam<br>1 cup tea, 1 tbsp sugar	**Lunch**<br>1 medium bowl white rice<br>¼ cup okra in tamarind gravy<br>¼ cup French beans & carrot<br>Chickpeas curry:<br>¼ cup chickpea<br>2 tbsp curry gravy<br>½ cup curd<br>1 cup tea, 1 tbsp sugar	**Lunch**<br>1 medium bowl white rice<br>¼ cup okra in tamarind gravy<br>¼ cup French beans & carrot<br>Chickpeas curry:<br>¼ cup chickpea<br>2 tbsp curry gravy<br>½ cup curd<br>1 cup tea, 1 tbsp sugar	**Lunch**<br>1 medium bowl white rice<br>¼ cup okra in tamarind gravy<br>¼ cup French beans & carrot<br>Chicken and chickpea curry:<br>½ oz chicken<br>¼ cup chickpea<br>2 tbsp curry gravy<br>1 small bowl rasam<br>1 cup tea, 1 tbsp sugar
**Evening snack**<br>1 pc apple<br>1 cup tea, 1 tbsp sugar	**Evening snack**<br>1 pc apple<br>1 cup tea, 1 tbsp sugar	**Evening snack**<br>1 pc apple<br>1 cup tea, 1 tbsp sugar	**Evening snack**<br>1 pc apple<br>1 cup tea, 1 tbsp sugar	**Evening snack**<br>1 pc apple<br>1 cup tea, 1 tbsp sugar	**Evening snack**<br>1 pc apple<br>1 cup tea, 1 tbsp sugar
**Dinner**<br>1 medium bowl white rice<br>¼ cup okra in tamarind gravy<br>½ cup stir-fried cabbage<br>1 oz deep fried fish<br>1 small bowl rasam<br>1 cup tea, 1 tbsp sugar	**Dinner**<br>1 medium bowl white rice<br>¼ cup okra in tamarind gravy<br>½ cup stir-fried cabbage<br>½ cup sambar<br>1 small bowl rasam<br>1 cup tea, 1 tbsp sugar	**Dinner**<br>1 medium bowl white rice<br>¼ cup okra in tamarind gravy<br>½ cup stir-fried cabbage<br>1 pc fried egg<br>1 small bowl rasam<br>1 cup tea, 1 tbsp sugar	**Dinner**<br>1 medium bowl white rice<br>¼ cup okra in tamarind gravy<br>½ cup stir-fried cabbage<br>¼ cup sambar<br>½ cup curd<br>1 cup tea, 1 tbsp sugar	**Dinner**<br>1 medium bowl white rice<br>¼ cup okra in tamarind gravy<br>½ cup stir-fried cabbage<br>1 pc fried egg<br>1 cup tea, 1 tbsp sugar	**Dinner**<br>1 medium bowl white rice<br>¼ cup okra in tamarind gravy<br>½ cup stir-fried cabbage<br>1 oz deep fried fish<br>1 small bowl rasam<br>1 cup tea, 1 tbsp sugar

DAY 2 (North Indian Menu)

Conventional	Vegan	Ovo-vegetarian	Lacto-vegetarian	Lacto-ovo vegetarian	Plant-dominant
**Breakfast**<br>1 bowl vermicelli upma<br>1 cup tea, 1 tbsp sugar	**Breakfast**<br>1 bowl vermicelli upma<br>1 cup tea, 1 tbsp sugar	**Breakfast**<br>1 bowl vermicelli upma<br>1 cup tea, 1 tbsp sugar	**Breakfast**<br>1 bowl vermicelli upma<br>1 cup tea, 1 tbsp sugar	**Breakfast**<br>1 bowl vermicelli upma<br>1 cup tea, 1 tbsp sugar	**Breakfast**<br>1 bowl vermicelli upma<br>1 cup tea, 1 tbsp sugar
**Morning snack**<br>1 pc orange<br>1 cup tea, 1 tbsp sugar	**Morning snack**<br>1 pc orange<br>1 cup tea, 1 tbsp sugar	**Morning snack**<br>1 pc orange<br>1 cup tea, 1 tbsp sugar	**Morning snack**<br>1 pc orange<br>1 cup tea, 1 tbsp sugar	**Morning snack**<br>1 pc orange<br>1 cup tea, 1 tbsp sugar	**Morning snack**<br>1 pc orange<br>1 cup tea, 1 tbsp sugar
**Lunch**<br>1 medium bowl white rice<br>1 oz masala chicken<br>½ cup okra kadai sabji<br>1 cup tea, 1 tbsp sugar	**Lunch**<br>1 medium bowl white rice<br>½ cup masala chickpeas<br>½ cup okra kadai sabji<br>1 cup tea, 1 tbsp sugar	**Lunch**<br>1 medium bowl white rice<br>1 pc masala egg<br>½ cup okra kadai sabji<br>1 cup tea, 1 tbsp sugar	**Lunch**<br>1 medium bowl white rice<br>¼ cup masala paneer<br>½ cup okra kadai sabji<br>1 cup tea, 1 tbsp sugar	**Lunch**<br>1 medium bowl white rice<br>¼ cup masala paneer<br>½ cup okra kadai sabji<br>1 cup tea, 1 tbsp sugar	**Lunch**<br>1 medium bowl white rice<br>½ oz masala chicken<br>¼ cup masala chickpeas<br>½ cup okra kadai sabji<br>1 cup tea, 1 tbsp sugar
**Evening snack**<br>1 slice papaya<br>1 cup tea, 1 tbsp sugar	**Evening snack**<br>1 slice papaya<br>1 cup tea, 1 tbsp sugar	**Evening snack**<br>1 slice papaya<br>1 cup tea, 1 tbsp sugar	**Evening snack**<br>1 slice papaya<br>1 cup tea, 1 tbsp sugar	**Evening snack**<br>1 slice papaya<br>1 cup tea, 1 tbsp sugar	**Evening snack**<br>1 slice papaya<br>1 cup tea, 1 tbsp sugar
**Dinner**<br>1 medium bowl white rice<br>1 oz chili fish<br>½ cup curry eggplant<br>½ cup okra kadai sabji<br>1 cup tea, 1 tbsp sugar	**Dinner**<br>1 medium bowl white rice<br>½ cup chili lentils<br>½ cup curry eggplant<br>½ cup okra kadai sabji<br>1 cup tea, 1 tbsp sugar	**Dinner**<br>1 medium bowl white rice<br>½ cup chili lentils<br>½ cup curry eggplant<br>½ cup okra kadai sabji<br>1 cup tea, 1 tbsp sugar	**Dinner**<br>1 medium bowl white rice<br>½ cup chili lentils<br>½ cup curry eggplant<br>½ cup okra kadai sabji<br>1 cup tea, 1 tbsp sugar	**Dinner**<br>1 medium bowl white rice<br>1 pc chili egg<br>½ cup curry eggplant<br>½ cup okra kadai sabji<br>1 cup tea, 1 tbsp sugar	**Dinner**<br>1 medium bowl white rice<br>1 oz chili fish<br>½ cup curry eggplant<br>½ cup okra kadai sabji<br>1 cup tea, 1 tbsp sugar

DAY 3 (South Chinese Menu)

	Conventional	Vegan	Ovo-vegetarian	Lacto-vegetarian	Lacto-ovo vegetarian	Plant-dominant
**Breakfast**	Rice noodles with pork<br>1 medium bowl rice noodles<br>½ oz minced pork<br>¼ cup bok choy<br>1 cup tea, 1 tbsp sugar	Rice noodles with tofu<br>1 medium bowl rice noodles<br>⅔ pc tofu<br>¼ cup bok choy<br>1 cup tea, 1 tbsp sugar	Rice noodles with tofu<br>1 medium bowl rice noodles<br>⅔ pc tofu<br>¼ cup bok choy<br>1 cup tea, 1 tbsp sugar	Rice noodles with tofu<br>1 medium bowl rice noodles<br>⅔ pc tofu<br>¼ cup bok choy<br>1 glass low fat milk	Rice noodles with tofu<br>1 medium bowl rice noodles<br>⅔ pc tofu<br>¼ cup bok choy<br>1 cup tea, 1 tbsp sugar<br>1 glass low fat milk	Rice noodles with tofu<br>1 medium bowl rice noodles<br>⅔ pc tofu<br>¼ cup bok choy<br>1 cup tea, 1 tbsp sugar
**Morning snack**	1 pc pear<br>1 cup tea, 1 tbsp sugar	1 pc pear<br>1 cup tea, 1 tbsp sugar	1 pc pear<br>1 cup tea, 1 tbsp sugar	1 pc pear<br>1 cup tea, 1 tbsp sugar	1 pc pear<br>1 cup tea, 1 tbsp sugar	1 pc pear<br>1 cup tea, 1 tbsp sugar
**Lunch**	1 medium bowl white rice<br>½ oz steamed fish<br>½ cup cucumber, stir-fried<br>1 cup tea, 1 tbsp sugar	1 medium bowl white rice<br>1 pc steamed tofu<br>½ cup cucumber, stir-fried<br>1 cup tea, 1 tbsp sugar	1 medium bowl white rice<br>1 pc steamed tofu<br>½ cup cucumber, stir-fried<br>1 cup tea, 1 tbsp sugar	1 medium bowl white rice<br>½ pc steamed tofu<br>½ cup cucumber, stir-fried<br>1 cup tea, 1 tbsp sugar	1 medium bowl white rice<br>⅓ pc steamed tofu<br>½ cup cucumber, stir-fried<br>1 cup tea, 1 tbsp sugar	1 medium bowl white rice<br>½ oz steamed fish<br>½ cup cucumber, stir-fried<br>1 cup tea, 1 tbsp sugar
**Evening snack**	1 pc apple<br>1 cup tea, 1 tbsp sugar	1 pc apple<br>1 cup tea, 1 tbsp sugar	1 pc apple<br>1 cup tea, 1 tbsp sugar	1 pc apple<br>1 cup tea, 1 tbsp sugar	1 pc apple<br>1 cup tea, 1 tbsp sugar	1 pc apple<br>1 cup tea, 1 tbsp sugar
**Dinner**	1 medium bowl white rice<br>1 oz sesame oil chicken sauce<br>½ cup spinach, stir-fried<br>½ cup carrot, stir-fried<br>1 cup tea, 1 tbsp sugar	1 medium bowl white rice<br>1 pc deep-fried tofu<br>½ cup carrot, stir-fried<br>½ cup spinach, stir-fried<br>1 cup tea, 1 tbsp sugar	1 medium bowl white rice<br>1 pc fried egg<br>½ cup carrot, stir-fried<br>½ cup spinach, stir-fried<br>1 cup tea, 1 tbsp sugar	1 medium bowl white rice<br>⅓ pc deep fried tofu<br>½ cup carrot, stir-fried<br>½ cup spinach, stir-fried<br>1 cup tea, 1 tbsp sugar	1 medium bowl white rice<br>1 pc fried egg<br>½ cup carrot, stir-fried<br>½ cup spinach, stir-fried<br>1 cup tea, 1 tbsp sugar	1 medium bowl white rice<br>1 oz sesame oil chicken sauce<br>½ cup carrot, stir-fried<br>½ cup spinach, stir-fried<br>1 cup tea, 1 tbsp sugar

*Sugar may be replaced with honey, syrup, candies, or jam as other food choices.

Sample Menu for Low Protein Diet (LPD) – 2100 kcal, 0.6 g/kg/d or 42 g/d protein

DAY 1 (South Indian Menu)

Conventional	Vegan	Ovo-vegetarian	Lacto-vegetarian	Lacto-ovo vegetarian	Plant-dominant
**Breakfast**<br>2 pc dosa<br>2 tbsp curry gravy<br>1 cup tea, 1 tbsp sugar	**Breakfast**<br>2 pc dosa<br>2 tbsp curry gravy<br>1 cup tea, 1 tbsp sugar	**Breakfast**<br>2 pc dosa<br>2 tbsp curry gravy<br>1 cup tea, 1 tbsp sugar	**Breakfast**<br>2 pc dosa<br>2 tbsp curry gravy<br>1 cup tea, 1 tbsp sugar	**Breakfast**<br>2 pc dosa<br>2 tbsp curry gravy<br>1 cup tea, 1 tbsp sugar	**Breakfast**<br>2 pc dosa<br>2 tbsp curry gravy<br>1 cup tea, 1 tbsp sugar
**Morning snack**<br>½ pc mango<br>1 cup tea, 1 tbsp sugar	**Morning snack**<br>½ pc mango	**Morning snack**<br>½ pc mango	**Morning snack**<br>½ pc mango<br>1 cup tea, 1 tbsp sugar	**Morning snack**<br>½ pc mango<br>1 cup tea, 1 tbsp sugar	**Morning snack**<br>½ pc mango<br>1 cup tea, 1 tbsp sugar
**Lunch**<br>1 medium bowl white rice<br>¼ cup okra in tamarind gravy<br>¼ cup french beans & carrot<br>Chicken curry:<br>1 oz chicken<br>2 tbsp curry gravy<br>1 small bowl rasam<br>1 cup tea, 1 tbsp sugar	**Lunch**<br>1 medium bowl white rice<br>¼ cup okra in tamarind gravy<br>¼ cup french beans & carrot<br>Chickpeas curry:<br>1 cup chickpea<br>2 tbsp curry gravy<br>1 small bowl rasam<br>1 cup tea, 1 tbsp sugar	**Lunch**<br>1 medium bowl white rice<br>¼ cup okra in tamarind gravy<br>¼ cup french beans & carrot<br>Chickpeas curry:<br>½ cup chickpea<br>2 tbsp curry gravy<br>1 small bowl rasam<br>1 cup tea, 1 tbsp sugar	**Lunch**<br>1 medium bowl white rice<br>¼ cup okra in tamarind gravy<br>¼ cup french beans & carrot<br>Chickpeas curry:<br>½ cup chickpea<br>2 tbsp curry gravy<br>1 cup curd<br>1 cup tea, 1 tbsp sugar	**Lunch**<br>1 medium bowl white rice<br>¼ cup okra in tamarind gravy<br>¼ cup french beans & carrot<br>Chickpeas curry:<br>¼ cup chickpea<br>2 tbsp curry gravy<br>1 cup curd<br>1 cup tea, 1 tbsp sugar	**Lunch**<br>1 medium bowl white rice<br>¼ cup okra in tamarind gravy<br>¼ cup french beans & carrot<br>Chicken and chickpea curry:<br>1 oz chicken<br>½ cup chickpea<br>2 tbsp curry gravy<br>1 small bowl rasam<br>1 cup tea, 1 tbsp sugar
**Evening snack**<br>1 pc apple<br>1 cup tea, 1 tbsp sugar	**Evening snack**<br>1 pc apple<br>1 cup tea, 1 tbsp sugar	**Evening snack**<br>1 pc apple<br>1 cup tea, 1 tbsp sugar	**Evening snack**<br>1 pc apple<br>1 cup tea, 1 tbsp sugar	**Evening snack**<br>1 pc apple<br>1 cup tea, 1 tbsp sugar	**Evening snack**<br>1 pc apple<br>1 cup tea, 1 tbsp sugar
**Dinner**<br>1 medium bowl white rice<br>¼ cup okra in tamarind gravy<br>½ cup stir-fried cabbage<br>2 oz deep fried fish<br>1 small bowl rasam<br>1 cup tea, 1 tbsp sugar	**Dinner**<br>1 medium bowl white rice<br>¼ cup okra in tamarind gravy<br>½ cup stir-fried cabbage<br>½ cup sambar<br>1 small bowl rasam<br>1 cup tea, 1 tbsp sugar	**Dinner**<br>1 medium bowl white rice<br>¼ cup okra in tamarind gravy<br>½ cup stir-fried cabbage<br>2 pc fried egg<br>1 small bowl rasam<br>1 cup tea, 1 tbsp sugar	**Dinner**<br>1 medium bowl white rice<br>¼ cup okra in tamarind gravy<br>½ cup stir-fried cabbage<br>¼ cup sambar<br>½ cup curd<br>1 cup tea, 1 tbsp sugar	**Dinner**<br>1 medium bowl white rice<br>¼ cup okra in tamarind gravy<br>½ cup stir-fried cabbage<br>2 pc fried egg<br>1 small bowl rasam<br>1 cup tea, 1 tbsp sugar	**Dinner**<br>1 medium bowl white rice<br>¼ cup okra in tamarind gravy<br>½ cup stir-fried cabbage<br>1 oz deep fried fish<br>1 small bowl rasam<br>1 cup tea, 1 tbsp sugar

DAY 2 (North Indian Menu)

Conventional	Vegan	Ovo-vegetarian	Lacto-vegetarian	Lacto-ovo vegetarian	Plant-dominant
**Breakfast**<br>1 bowl vermicelli upma<br>1 cup tea, 1 tbsp sugar	**Breakfast**<br>1 bowl vermicelli upma<br>1 cup tea, 1 tbsp sugar	**Breakfast**<br>1 bowl vermicelli upma<br>1 cup tea, 1 tbsp sugar	**Breakfast**<br>1 bowl vermicelli upma<br>1 cup tea, 1 tbsp sugar	**Breakfast**<br>1 bowl vermicelli upma<br>1 cup tea, 1 tbsp sugar	**Breakfast**<br>1 bowl vermicelli upma<br>1 cup tea, 1 tbsp sugar
**Morning snack**<br>1 pc orange<br>1 cup tea, 1 tbsp sugar	**Morning snack**<br>1 pc orange	**Morning snack**<br>1 pc orange	**Morning snack**<br>1 pc orange	**Morning snack**<br>1 pc orange<br>1 cup tea, 1 tbsp sugar	**Morning snack**<br>1 pc orange<br>1 cup tea, 1 tbsp sugar
**Lunch**<br>1 medium bowl white rice<br>1 oz masala chicken<br>½ cup okra kadai sabji<br>1 cup tea, 1 tbsp sugar	**Lunch**<br>1 medium bowl white rice<br>½ cup masala chickpeas<br>½ cup okra kadai sabji<br>1 cup tea, 1 tbsp sugar	**Lunch**<br>1 medium bowl white rice<br>2 pc masala egg<br>½ cup okra kadai sabji<br>1 cup tea, 1 tbsp sugar	**Lunch**<br>1 medium bowl white rice<br>½ cup masala paneer<br>½ cup okra kadai sabji<br>1 cup tea, 1 tbsp sugar	**Lunch**<br>1 medium bowl white rice<br>¼ cup masala paneer<br>½ cup okra kadai sabji<br>1 cup tea, 1 tbsp sugar	**Lunch**<br>1 medium bowl white rice<br>1 oz masala chicken<br>½ cup masala chickpeas<br>½ cup okra kadai sabji<br>1 cup tea, 1 tbsp sugar
**Evening snack**<br>1 slice papaya<br>1 cup tea, 1 tbsp sugar	**Evening snack**<br>1 slice papaya<br>1 cup tea, 1 tbsp sugar	**Evening snack**<br>1 slice papaya<br>1 cup tea, 1 tbsp sugar	**Evening snack**<br>1 slice papaya<br>1 cup tea, 1 tbsp sugar	**Evening snack**<br>1 slice papaya<br>1 cup tea, 1 tbsp sugar	**Evening snack**<br>1 slice papaya<br>1 cup tea, 1 tbsp sugar
**Dinner**<br>1 medium bowl white rice<br>2 oz chili fish<br>½ cup curry eggplant<br>½ cup okra kadai sabji<br>1 cup tea, 1 tbsp sugar	**Dinner**<br>1 medium bowl white rice<br>1 cup chili lentils<br>½ cup curry eggplant<br>½ cup okra kadai sabji<br>1 cup tea, 1 tbsp sugar	**Dinner**<br>1 medium bowl white rice<br>½ cup chili lentils<br>½ cup curry eggplant<br>½ cup okra kadai sabji<br>1 cup tea, 1 tbsp sugar	**Dinner**<br>1 medium bowl white rice<br>½ cup chili lentils<br>½ cup curry eggplant<br>½ cup okra kadai sabji<br>1 cup tea, 1 tbsp sugar	**Dinner**<br>1 medium bowl white rice<br>2 pc chili egg<br>½ cup curry eggplant<br>½ cup okra kadai sabji<br>1 cup tea, 1 tbsp sugar	**Dinner**<br>1 medium bowl white rice<br>1 oz chili fish<br>½ cup curry eggplant<br>½ cup okra kadai sabji<br>1 cup tea, 1 tbsp sugar

DAY 3 (South Chinese Menu)

Conventional	Vegan	Ovo-vegetarian	Lacto-vegetarian	Lacto-ovo vegetarian	Plant-dominant
**Breakfast**<br>Rice noodles with pork<br>1 medium bowl rice noodles<br>½ oz minced pork<br>¼ cup bok choy<br>1 cup tea, 1 tbsp sugar	**Breakfast**<br>Rice noodles with tofu<br>1 medium bowl rice noodles<br>1 pc tofu<br>¼ cup bok choy<br>1 cup tea, 1 tbsp sugar	**Breakfast**<br>Rice noodles with tofu<br>1 medium bowl rice noodles<br>⅔ pc tofu<br>¼ cup bok choy<br>1 cup tea, 1 tbsp sugar	**Breakfast**<br>Rice noodles with tofu<br>1 medium bowl rice noodles<br>⅔ pc tofu<br>¼ cup bok choy<br>1 glass low fat milk	**Breakfast**<br>Rice noodles with tofu<br>1 medium bowl rice noodles<br>⅔ pc tofu<br>¼ cup bok choy<br>1 glass low fat milk	**Breakfast**<br>Rice noodles with tofu<br>1 medium bowl rice noodles<br>1 pc tofu<br>¼ cup bok choy<br>1 cup tea, 1 tbsp sugar
**Morning snack**<br>1 pc pear<br>1 cup tea, 1 tbsp sugar	**Morning snack**<br>1 pc pear	**Morning snack**<br>1 pc pear	**Morning snack**<br>1 pc pear<br>1 cup tea, 1 tbsp sugar	**Morning snack**<br>1 pc pear<br>1 cup tea, 1 tbsp sugar	**Morning snack**<br>1 pc pear<br>1 cup tea, 1 tbsp sugar
**Lunch**<br>1 medium bowl white rice<br>1½ oz steamed fish<br>½ cup cucumber, stir-fried<br>1 cup tea, 1 tbsp sugar	**Lunch**<br>1 medium bowl white rice<br>1 pc steamed tofu<br>½ cup cucumber, stir-fried<br>1 cup tea, 1 tbsp sugar	**Lunch**<br>1 medium bowl white rice<br>1 pc steamed tofu<br>½ cup cucumber, stir-fried<br>1 cup tea, 1 tbsp sugar	**Lunch**<br>1 medium bowl white rice<br>1 pc steamed tofu<br>½ cup cucumber, stir-fried<br>1 cup tea, 1 tbsp sugar	**Lunch**<br>1 medium bowl white rice<br>⅓ pc steamed tofu<br>½ cup cucumber, stir-fried<br>1 cup tea, 1 tbsp sugar	**Lunch**<br>1 medium bowl white rice<br>1 oz steamed fish<br>½ cup cucumber, stir-fried<br>1 cup tea, 1 tbsp sugar
**Evening snack**<br>1 pc apple<br>1 cup tea, 1 tbsp sugar	**Evening snack**<br>1 pc apple<br>1 cup tea, 1 tbsp sugar	**Evening snack**<br>1 pc apple<br>1 cup tea, 1 tbsp sugar	**Evening snack**<br>1 pc apple<br>1 cup tea, 1 tbsp sugar	**Evening snack**<br>1 pc apple<br>1 cup tea, 1 tbsp sugar	**Evening snack**<br>1 pc apple<br>1 cup tea, 1 tbsp sugar
**Dinner**<br>1 medium bowl white rice<br>1 oz sesame oil chicken sauce<br>½ cup carrot, stir-fried<br>½ cup spinach, stir-fried<br>1 cup tea, 1 tbsp sugar	**Dinner**<br>1 medium bowl white rice<br>3 oz deep fried tofu<br>½ cup carrot, stir-fried<br>½ cup spinach, stir-fried<br>1 cup tea, 1 tbsp sugar	**Dinner**<br>1 medium bowl white rice<br>2 pc fried egg<br>½ cup carrot, stir-fried<br>½ cup spinach, stir-fried<br>1 cup tea, 1 tbsp sugar	**Dinner**<br>1 medium bowl white rice<br>⅔ pc deep fried tofu<br>½ cup carrot, stir-fried<br>½ cup spinach, stir-fried<br>1 cup tea, 1 tbsp sugar	**Dinner**<br>1 medium bowl white rice<br>2 pc fried egg<br>½ cup carrot, stir-fried<br>½ cup spinach, stir-fried<br>1 cup tea, 1 tbsp sugar	**Dinner**<br>1 medium bowl white rice<br>3 oz deep fried tofu<br>½ cup carrot, stir-fried<br>½ cup spinach, stir-fried<br>1 cup tea, 1 tbsp sugar

*Sugar may be replaced with honey, syrup, candies, or jam as other food choices.

Sample Menu for Low Protein Diet (LPD) – 2100 kcal, 0.7 g/kg/d or 49 g/d protein

DAY 1 (South Indian Menu)

Conventional	Vegan	Ovo-vegetarian	Lacto-vegetarian	Lacto-ovo vegetarian	Plant-dominant
**Breakfast**<br>2 pc dosa<br>2 tbsp curry gravy<br>1 cup tea, 1 tbsp sugar	**Breakfast**<br>2 pc dosa<br>2 tbsp curry gravy<br>1 cup tea, 1 tbsp sugar	**Breakfast**<br>2 pc dosa<br>2 tbsp curry gravy<br>1 cup tea, 1 tbsp sugar	**Breakfast**<br>2 pc dosa<br>2 tbsp curry gravy<br>1 cup tea, 1 tbsp sugar	**Breakfast**<br>2 pc dosa<br>2 tbsp curry gravy<br>1 cup tea, 1 tbsp sugar	**Breakfast**<br>2 pc dosa<br>2 tbsp curry gravy<br>1 cup tea, 1 tbsp sugar
**Morning snack**<br>½ pc mango	**Morning snack**<br>½ pc mango	**Morning snack**<br>½ pc mango	**Morning snack**<br>½ pc mango	**Morning snack**<br>½ pc mango	**Morning snack**<br>½ pc mango
**Lunch**<br>1 medium bowl white rice<br>¼ cup okra in tamarind gravy<br>¼ cup french beans & carrot<br>Chicken curry:<br>2 oz chicken<br>2 tbsp curry gravy<br>1 small bowl rasam<br>1 cup tea, 1 tbsp sugar	**Lunch**<br>1 medium bowl white rice<br>¼ cup okra in tamarind gravy<br>¼ cup french beans & carrot<br>Chickpeas curry:<br>1 cup chickpea<br>2 tbsp curry gravy<br>1 small bowl rasam<br>1 cup tea, 1 tbsp sugar	**Lunch**<br>1 medium bowl white rice<br>¼ cup okra in tamarind gravy<br>¼ cup french beans & carrot<br>Chickpeas curry:<br>1 cup chickpea<br>2 tbsp curry gravy<br>1 small bowl rasam<br>1 cup tea, 1 tbsp sugar	**Lunch**<br>1 medium bowl white rice<br>¼ cup okra in tamarind gravy<br>¼ cup french beans & carrot<br>Chickpeas curry:<br>1 cup chickpea<br>2 tbsp curry gravy<br>1 cup curd<br>1 cup tea, 1 tbsp sugar	**Lunch**<br>1 medium bowl white rice<br>¼ cup okra in tamarind gravy<br>¼ cup french beans & carrot<br>Chickpeas curry:<br>½ cup chickpea<br>2 tbsp curry gravy<br>½ cup curd<br>1 cup tea, 1 tbsp sugar	**Lunch**<br>1 medium bowl white rice<br>¼ cup okra in tamarind gravy<br>¼ cup french beans & carrot<br>Chicken and chickpea curry:<br>1 oz chicken<br>1 cup chickpea<br>2 tbsp curry gravy<br>1 small bowl rasam<br>1 cup tea, 1 tbsp sugar
**Evening snack**<br>1 pc apple<br>1 cup tea, 1 tbsp sugar	**Evening snack**<br>1 pc apple	**Evening snack**<br>1 pc apple	**Evening snack**<br>1 pc apple	**Evening snack**<br>1 pc apple<br>1 cup tea, 1 tbsp sugar	**Evening snack**<br>1 pc apple<br>1 cup tea, 1 tbsp sugar
**Dinner**<br>1 medium bowl white rice<br>¼ cup okra in tamarind gravy<br>½ cup stir-fried cabbage<br>2 oz deep fried fish<br>1 small bowl rasam<br>1 cup tea, 1 tbsp sugar	**Dinner**<br>1 medium bowl white rice<br>¼ cup okra in tamarind gravy<br>½ cup stir-fried cabbage<br>1 cup sambar<br>1 small bowl rasam<br>1 cup tea, 1 tbsp sugar	**Dinner**<br>1 medium bowl white rice<br>¼ cup okra in tamarind gravy<br>½ cup stir-fried cabbage<br>2 pc fried egg<br>1 small bowl rasam<br>1 cup tea, 1 tbsp sugar	**Dinner**<br>1 medium bowl white rice<br>¼ cup okra in tamarind gravy<br>½ cup stir-fried cabbage<br>¼ cup sambar<br>½ cup curd<br>1 cup tea, 1 tbsp sugar	**Dinner**<br>1 medium bowl white rice<br>¼ cup okra in tamarind gravy<br>½ cup stir-fried cabbage<br>2 pc fried egg<br>½ cup curd<br>1 cup tea, 1 tbsp sugar	**Dinner**<br>1 medium bowl white rice<br>¼ cup okra in tamarind gravy<br>½ cup stir-fried cabbage<br>1 oz deep fried fish<br>1 small bowl rasam<br>1 cup tea, 1 tbsp sugar

DAY 2 (North Indian Menu)

Conventional	Vegan	Ovo-vegetarian	Lacto-vegetarian	Lacto-ovo vegetarian	Plant-dominant
**Breakfast**<br>1 bowl vermicelli upma<br>1 cup tea, 1 tbsp sugar	**Breakfast**<br>1 bowl vermicelli upma<br>1 cup tea, 1 tbsp sugar	**Breakfast**<br>1 bowl vermicelli upma<br>1 cup tea, 1 tbsp sugar	**Breakfast**<br>1 bowl vermicelli upma<br>1 cup tea, 1 tbsp sugar	**Breakfast**<br>1 bowl vermicelli upma<br>1 cup tea, 1 tbsp sugar	**Breakfast**<br>1 bowl vermicelli upma<br>1 cup tea, 1 tbsp sugar
**Morning snack**<br>1 pc orange<br>1 cup tea, 1 tbsp sugar	**Morning snack**<br>1 pc orange	**Morning snack**<br>1 pc orange	**Morning snack**<br>1 pc orange	**Morning snack**<br>1 pc orange	**Morning snack**<br>1 pc orange
**Lunch**<br>1 medium bowl white rice<br>2 oz masala chicken<br>½ cup okra kadai sabji<br>1 cup tea, 1 tbsp sugar	**Lunch**<br>1 medium bowl white rice<br>1 cup masala chickpeas<br>½ cup okra kadai sabji<br>1 cup tea, 1 tbsp sugar	**Lunch**<br>1 medium bowl white rice<br>2 pc masala egg<br>½ cup okra kadai sabji<br>1 cup tea, 1 tbsp sugar	**Lunch**<br>1 medium bowl white rice<br>½ cup masala paneer<br>½ cup okra kadai sabji<br>1 cup tea, 1 tbsp sugar	**Lunch**<br>1 medium bowl white rice<br>½ cup masala paneer<br>½ cup okra kadai sabji<br>1 cup tea, 1 tbsp sugar	**Lunch**<br>1 medium bowl white rice<br>1 oz masala chicken<br>1 cup masala chickpeas<br>½ cup okra kadai sabji<br>1 cup tea, 1 tbsp sugar
**Evening snack**<br>1 slice papaya<br>1 cup tea, 1 tbsp sugar	**Evening snack**<br>1 slice papaya	**Evening snack**<br>1 slice papaya	**Evening snack**<br>1 slice papaya	**Evening snack**<br>1 slice papaya<br>1 cup tea, 1 tbsp sugar	**Evening snack**<br>1 slice papaya<br>1 cup tea, 1 tbsp sugar
**Dinner**<br>1 medium bowl white rice<br>2 oz chili fish<br>½ cup curry eggplant<br>½ cup okra kadai sabji<br>1 cup tea, 1 tbsp sugar	**Dinner**<br>1 medium bowl white rice<br>1 cup chili lentils<br>½ cup curry eggplant<br>½ cup okra kadai sabji<br>1 cup tea, 1 tbsp sugar	**Dinner**<br>1 medium bowl white rice<br>1 cup chili lentils<br>½ cup curry eggplant<br>½ cup okra kadai sabji<br>1 cup tea, 1 tbsp sugar	**Dinner**<br>1 medium bowl white rice<br>1 cup chili lentils<br>½ cup curry eggplant<br>½ cup okra kadai sabji<br>1 cup tea, 1 tbsp sugar	**Dinner**<br>1 medium bowl white rice<br>2 pc chili egg<br>½ cup curry eggplant<br>½ cup okra kadai sabji<br>1 cup tea, 1 tbsp sugar	**Dinner**<br>1 medium bowl white rice<br>1 oz chili fish<br>½ cup curry eggplant<br>½ cup okra kadai sabji<br>1 cup tea, 1 tbsp sugar

DAY 3 (South Chinese Menu)

Conventional	Vegan	Ovo-vegetarian	Lacto-vegetarian	Lacto-ovo vegetarian	Plant-dominant
**Breakfast**<br>Rice noodles with pork<br>1 medium bowl rice noodles<br>½ oz minced pork<br>¼ cup bok choy<br>1 cup tea, 1 tbsp sugar	**Breakfast**<br>Rice noodles with tofu<br>1 medium bowl rice noodles<br>1⅓ pc tofu<br>¼ cup bok choy<br>1 cup tea, 1 tbsp sugar	**Breakfast**<br>Rice noodles with tofu<br>1 medium bowl rice noodles<br>1 pc tofu<br>¼ cup bok choy<br>1 cup tea, 1 tbsp sugar	**Breakfast**<br>Rice noodles with tofu<br>1 medium bowl rice noodles<br>⅔ pc tofu<br>¼ cup bok choy<br>1 glass low fat milk	**Breakfast**<br>Rice noodles with tofu<br>1 medium bowl rice noodles<br>⅔ pc tofu<br>¼ cup bok choy<br>1 glass low fat milk	**Breakfast**<br>Rice noodles with tofu<br>1 medium bowl rice noodles<br>1 pc tofu<br>¼ cup bok choy<br>1 cup tea, 1 tbsp sugar
**Morning snack**<br>1 pc pear<br>1 cup tea, 1 tbsp sugar	**Morning snack**<br>1 pc pear	**Morning snack**<br>1 pc pear	**Morning snack**<br>1 pc pear<br>½ glass low fat milk	**Morning snack**<br>1 pc pear<br>½ glass low fat milk	**Morning snack**<br>1 pc pear
**Lunch**<br>1 medium bowl white rice<br>1½ oz steamed fish<br>½ cup cucumber, stir-fried<br>1 cup tea, 1 tbsp sugar	**Lunch**<br>1 medium bowl white rice<br>1⅓ pc steamed tofu<br>½ cup cucumber, stir-fried<br>1 cup tea, 1 tbsp sugar	**Lunch**<br>1 medium bowl white rice<br>1⅓ pc steamed tofu<br>½ cup cucumber, stir-fried<br>1 cup tea, 1 tbsp sugar	**Lunch**<br>1 medium bowl white rice<br>3 oz steamed tofu<br>½ cup cucumber, stir-fried<br>1 cup tea, 1 tbsp sugar	**Lunch**<br>1 medium bowl white rice<br>1 oz steamed tofu<br>½ cup cucumber, stir-fried<br>1 cup tea, 1 tbsp sugar	**Lunch**<br>1 medium bowl white rice<br>1 oz steamed fish<br>½ cup cucumber, stir-fried<br>1 cup tea, 1 tbsp sugar
**Evening snack**<br>1 pc apple<br>1 cup tea, 1 tbsp sugar	**Evening snack**<br>1 pc apple	**Evening snack**<br>1 pc apple	**Evening snack**<br>1 pc apple<br>½ glass low fat milk	**Evening snack**<br>1 pc apple<br>½ glass low fat milk	**Evening snack**<br>1 pc apple<br>1 cup tea, 1 tbsp sugar
**Dinner**<br>1 medium bowl white rice<br>1½ oz sesame oil chicken sauce<br>½ cup carrot, stir-fried<br>½ cup spinach, stir-fried<br>1 cup tea, 1 tbsp sugar	**Dinner**<br>1 medium bowl white rice<br>1⅓ pc deep fried tofu<br>½ cup carrot, stir-fried<br>½ cup spinach, stir-fried<br>1 cup tea, 1 tbsp sugar	**Dinner**<br>1 medium bowl white rice<br>2 pc fried egg<br>½ cup carrot, stir-fried<br>½ cup spinach, stir-fried<br>1 cup tea, 1 tbsp sugar	**Dinner**<br>1 medium bowl white rice<br>⅓ pc deep fried tofu<br>½ cup carrot, stir-fried<br>½ cup spinach, stir-fried<br>1 cup tea, 1 tbsp sugar	**Dinner**<br>1 medium bowl white rice<br>2 pc fried egg<br>½ cup carrot, stir-fried<br>½ cup spinach, stir-fried<br>1 cup tea, 1 tbsp sugar	**Dinner**<br>1 medium bowl white rice<br>1 pc deep fried tofu<br>1 oz sesame oil chicken sauce<br>½ cup carrot, stir-fried<br>½ cup spinach, stir-fried<br>1 cup tea, 1 tbsp sugar

*Sugar may be replaced with honey, syrup, candies, or jam as other food choices.

Sample Menu for Low Protein Diet (LPD) – 2100 kcal, 0.8 g/kg/d or 56 g/d protein

DAY 1 (South Indian Menu)

Conventional	Vegan	Ovo-vegetarian	Lacto-vegetarian	Lacto-ovo vegetarian	Plant-dominant
**Breakfast**<br>2 pc dosa<br>2 tbsp curry gravy<br>1 cup tea, 1 tbsp sugar	**Breakfast**<br>2 pc dosa<br>2 tbsp curry gravy<br>1 cup tea, 1 tbsp sugar	**Breakfast**<br>2 pc dosa<br>2 tbsp curry gravy<br>1 cup tea, 1 tbsp sugar	**Breakfast**<br>2 pc dosa<br>2 tbsp curry gravy<br>1 cup tea, 1 tbsp sugar	**Breakfast**<br>2 pc dosa<br>2 tbsp curry gravy<br>1 cup tea, 1 tbsp sugar	**Breakfast**<br>2 pc dosa<br>2 tbsp curry gravy<br>1 cup tea, 1 tbsp sugar
**Morning snack**<br>½ pc mango	**Morning snack**<br>½ pc mango	**Morning snack**<br>½ pc mango	**Morning snack**<br>½ pc mango	**Morning snack**<br>½ pc mango	**Morning snack**<br>½ pc mango
**Lunch**<br>1 medium bowl white rice<br>¼ cup okra in tamarind gravy<br>¼ cup french beans & carrot<br>Chicken curry:<br>2 oz chicken<br>2 tbsp curry gravy<br>1 small bowl rasam<br>1 cup tea, 1 tbsp sugar	**Lunch**<br>1 medium bowl white rice<br>¼ cup okra in tamarind gravy<br>¼ cup french beans & carrot<br>Chickpeas curry:<br>1½ cup chickpea<br>2 tbsp curry gravy<br>1 small bowl rasam	**Lunch**<br>1 medium bowl white rice<br>¼ cup okra in tamarind gravy<br>¼ cup french beans & carrot<br>Chickpeas curry:<br>1 cup chickpea<br>2 tbsp curry gravy<br>1 small bowl rasam	**Lunch**<br>1 medium bowl white rice<br>¼ cup okra in tamarind gravy<br>¼ cup french beans & carrot<br>Chickpeas curry:<br>1 cup chickpea<br>2 tbsp curry gravy<br>1 cup curd	**Lunch**<br>1 medium bowl white rice<br>¼ cup okra in tamarind gravy<br>¼ cup french beans & carrot<br>Chickpeas curry:<br>1 cup chickpea<br>2 tbsp curry gravy<br>½ cup curd	**Lunch**<br>1 medium bowl white rice<br>¼ cup okra in tamarind gravy<br>¼ cup french beans & carrot<br>Chicken and chickpea curry:<br>1 oz chicken<br>1 cup chickpea<br>2 tbsp curry gravy<br>1 small bowl rasam
**Evening snack**<br>1 pc apple	**Evening snack**<br>1 pc apple	**Evening snack**<br>1 pc apple	**Evening snack**<br>1 pc apple	**Evening snack**<br>1 pc apple	**Evening snack**<br>1 pc apple
**Dinner**<br>1 medium bowl white rice<br>¼ cup okra in tamarind gravy<br>½ cup stir-fried cabbage<br>2½ oz deep fried fish<br>1 small bowl rasam<br>1 cup tea, 1 tbsp sugar	**Dinner**<br>1 medium bowl white rice<br>¼ cup okra in tamarind gravy<br>½ cup stir-fried cabbage<br>1 cup sambar<br>1 small bowl rasam<br>1 cup tea, 1 tbsp sugar	**Dinner**<br>1 medium bowl white rice<br>¼ cup okra in tamarind gravy<br>½ cup stir-fried cabbage<br>2 pc fried egg<br>½ cup sambar<br>1 small bowl rasam<br>1 cup tea, 1 tbsp sugar	**Dinner**<br>1 medium bowl white rice<br>¼ cup okra in tamarind gravy<br>½ cup stir-fried cabbage<br>½ cup sambar<br>½ cup curd<br>1 cup tea, 1 tbsp sugar	**Dinner**<br>1 medium bowl white rice<br>¼ cup okra in tamarind gravy<br>½ cup stir-fried cabbage<br>2 pc fried egg<br>½ cup curd<br>1 cup tea, 1 tbsp sugar	**Dinner**<br>1 medium bowl white rice<br>¼ cup okra in tamarind gravy<br>½ cup stir-fried cabbage<br>2 oz deep fried fish<br>1 small bowl rasam<br>1 cup tea, 1 tbsp sugar

DAY 2 (North Indian Menu)

	Conventional	Vegan	Ovo-vegetarian	Lacto-vegetarian	Lacto-ovo vegetarian	Plant-dominant
**Breakfast**	1 bowl vermicelli upma<br>1 cup tea, 1 tbsp sugar	1 bowl vermicelli upma<br>1 cup tea, 1 tbsp sugar	1 bowl vermicelli upma<br>1 cup tea, 1 tbsp sugar	1 bowl vermicelli upma<br>1 cup tea, 1 tbsp sugar	1 bowl vermicelli upma<br>1 cup tea, 1 tbsp sugar	1 bowl vermicelli upma<br>1 cup tea, 1 tbsp sugar
**Morning snack**	1 pc orange<br>1 cup tea, 1 tbsp sugar	1 pc orange	1 pc orange	1 pc orange	1 pc orange	1 pc orange
**Lunch**	1 medium bowl white rice<br>2½ oz masala chicken<br>½ cup okra kadai sabji<br>1 cup tea, 1 tbsp sugar	1 medium bowl white rice<br>1 cup masala chickpeas<br>½ cup okra kadai sabji	1 medium bowl white rice<br>2 pc masala egg<br>½ cup masala chickpeas<br>½ cup okra kadai sabji	1 medium bowl white rice<br>½ cup masala paneer<br>½ cup masala chickpeas<br>½ cup okra kadai sabji	1 medium bowl white rice<br>½ cup masala paneer<br>½ cup okra kadai sabji	1 medium bowl white rice<br>1 oz masala chicken<br>1 cup masala chickpeas<br>½ cup okra kadai sabji
**Evening snack**	1 slice papaya	1 slice papaya	1 slice papaya	1 slice papaya	1 slice papaya	1 slice papaya
**Dinner**	1 medium bowl white rice<br>2 oz chili fish<br>½ cup curry eggplant<br>½ cup okra kadai sabji<br>1 cup tea, 1 tbsp sugar	1 medium bowl white rice<br>1½ cup chili lentils<br>½ cup curry eggplant<br>½ cup okra kadai sabji<br>1 cup tea, 1 tbsp sugar	1 medium bowl white rice<br>1 cup chili lentils<br>½ cup curry eggplant<br>½ cup okra kadai sabji<br>1 cup tea, 1 tbsp sugar	1 medium bowl white rice<br>1 cup chili lentils<br>½ cup curry eggplant<br>½ cup okra kadai sabji<br>1 cup tea, 1 tbsp sugar	1 medium bowl white rice<br>2 pc chili egg<br>½ cup chili lentils<br>½ cup curry eggplant<br>½ cup okra kadai sabji<br>1 cup tea, 1 tbsp sugar	1 medium bowl white rice<br>1 oz chili fish<br>½ cup chili lentils<br>½ cup curry eggplant<br>½ cup okra kadai sabji<br>1 cup tea, 1 tbsp sugar

DAY 3 (South Chinese Menu)

	Conventional	Vegan	Ovo-vegetarian	Lacto-vegetarian	Lacto-ovo vegetarian	Plant-dominant
**Breakfast**	Rice noodles with pork<br>1 medium bowl rice noodles<br>2 oz minced pork<br>¼ cup bok choy<br>1 cup tea, 1 tbsp sugar	Rice noodles with tofu<br>1 medium bowl rice noodles<br>1⅓ pc tofu<br>¼ cup bok choy<br>1 cup tea, 1 tbsp sugar	Rice noodles with tofu<br>1 medium bowl rice noodles<br>1 pc tofu<br>¼ cup bok choy<br>1 cup tea, 1 tbsp sugar	Rice noodles with tofu<br>1 medium bowl rice noodles<br>1 pc tofu<br>¼ cup bok choy<br>1 glass low fat milk	Rice noodles with tofu<br>1 medium bowl rice noodles<br>⅔ pc tofu<br>¼ cup bok choy<br>1 glass low fat milk	Rice noodles with tofu<br>1 medium bowl rice noodles<br>1 pc tofu<br>¼ cup bok choy<br>1 cup tea, 1 tbsp sugar
**Morning snack**	1 pc pear<br>1 cup tea, 1 tbsp sugar	1 pc pear	1 pc pear	1 pc pear<br>½ glass low fat milk	1 pc pear<br>½ glass low fat milk	1 pc pear
**Lunch**	1 medium bowl white rice<br>1½ oz steamed fish<br>½ cup cucumber, stir-fried<br>1 cup tea, 1 tbsp sugar	1 medium bowl white rice<br>2 pc steamed tofu<br>½ cup cucumber, stir-fried	1 medium bowl white rice<br>3 oz steamed tofu<br>½ cup cucumber, stir-fried	1 medium bowl white rice<br>1 pc steamed tofu<br>½ cup cucumber, stir-fried	1 medium bowl white rice<br>2/3 pc steamed tofu<br>½ cup cucumber, stir-fried	1 medium bowl white rice<br>1 oz steamed fish<br>1/3 pc steamed tofu<br>½ cup cucumber, stir-fried<br>1 cup tea, 1 tbsp sugar
**Evening snack**	1 pc apple	1 pc apple	1 pc apple	1 pc apple<br>½ glass low fat milk	1 pc apple<br>½ glass low fat milk	1 pc apple
**Dinner**	1 medium bowl white rice<br>1½ oz sesame oil chicken sauce<br>½ cup carrot, stir-fried<br>½ cup spinach, stir-fried<br>1 cup tea, 1 tbsp sugar	1 medium bowl white rice<br>1⅓ pc deep fried tofu<br>½ cup carrot, stir-fried<br>½ cup spinach, stir-fried<br>1 cup tea, 1 tbsp sugar	1 medium bowl white rice<br>2 pc fried egg<br>3 oz fried tofu<br>½ cup carrot, stir-fried<br>½ cup spinach, stir-fried<br>1 cup tea, 1 tbsp sugar	1 medium bowl white rice<br>1 pc deep fried tofu<br>½ cup carrot, stir-fried<br>½ cup spinach, stir-fried<br>1 cup tea, 1 tbsp sugar	1 medium bowl white rice<br>2 pc fried egg<br>½ cup carrot, stir-fried<br>½ cup spinach, stir-fried<br>1 cup tea, 1 tbsp sugar	1 medium bowl white rice<br>1 pc deep fried tofu<br>1 oz sesame oil chicken sauce<br>½ cup carrot, stir-fried<br>½ cup spinach, stir-fried<br>1 cup tea, 1 tbsp sugar

*Sugar may be replaced with honey, syrup, candies, or jam as other food choices.

Sample Menu for Moderate Protein Intake – 2100 kcal, 1.0 g/kg/d or 70 g/d protein

DAY 1 (South Indian Menu)

	Conventional	Vegan	Ovo-vegetarian	Lacto-vegetarian	Lacto-ovo vegetarian	Plant-dominant
**Breakfast**	3 pc dosa<br>2 tbsp curry gravy<br>1 cup tea, 1 tbsp sugar	3 pc dosa<br>2 tbsp curry gravy	3 pc dosa<br>2 tbsp curry gravy	3 pc dosa<br>2 tbsp curry gravy	3 pc dosa<br>2 tbsp curry gravy	3 pc dosa<br>2 tbsp curry gravy
**Morning snack**	3 pc cream crackers	3 pc cream crackers	3 pc cream crackers	3 pc cream crackers	3 pc cream crackers	3 pc cream crackers
**Lunch**	1 medium bowl white rice<br>½ cup okra in tamarind gravy<br>½ cup french beans & carrot<br>Chicken curry:<br>2 oz chicken<br>2 tbsp curry gravy<br>1 small bowl rasam<br>½ pc mango	1 medium bowl white rice<br>½ cup okra in tamarind gravy<br>½ cup french beans & carrot<br>Chickpeas curry:<br>1½ cup chickpea<br>2 tbsp curry gravy<br>1 small bowl rasam<br>½ pc mango	1 medium bowl white rice<br>½ cup okra in tamarind gravy<br>½ cup french beans & carrot<br>Chickpeas curry:<br>1 cup chickpea<br>2 tbsp curry gravy<br>1 small bowl rasam<br>½ pc mango	1 medium bowl white rice<br>½ cup okra in tamarind gravy<br>½ cup french beans & carrot<br>Chickpeas curry:<br>1 cup chickpea<br>2 tbsp curry gravy<br>½ cup curd<br>½ pc mango	1 medium bowl white rice<br>½ cup okra in tamarind gravy<br>½ cup french beans & carrot<br>Chickpeas curry:<br>1 cup chickpea<br>2 tbsp curry gravy<br>½ cup curd<br>½ pc mango	1 medium bowl white rice<br>½ cup okra in tamarind gravy<br>½ cup french beans & carrot<br>Chicken and chickpea curry:<br>1 oz chicken<br>½ cup chickpea<br>2 tbsp curry gravy<br>1 small bowl rasam<br>½ pc mango
**Evening snack**	1 pc apple	1 pc apple	1 pc apple	1 pc apple	1 pc apple	1 pc apple
**Dinner**	1 medium bowl white rice<br>¼ cup okra in tamarind gravy<br>½ cup stir-fried cabbage<br>2½ oz deep fried fish<br>1 small bowl rasam	1 medium bowl white rice<br>¼ cup okra in tamarind gravy<br>½ cup stir-fried cabbage<br>1 cup sambar<br>1 small bowl rasam	1 medium bowl white rice<br>¼ cup okra in tamarind gravy<br>½ cup stir-fried cabbage<br>2 pc fried egg<br>½ cup sambar<br>1 small bowl rasam	1 medium bowl white rice<br>¼ cup okra in tamarind gravy<br>½ cup stir-fried cabbage<br>½ cup sambar<br>½ cup curd	1 medium bowl white rice<br>¼ cup okra in tamarind gravy<br>½ cup stir-fried cabbage<br>2 pc fried egg<br>½ cup curd	1 medium bowl white rice<br>¼ cup okra in tamarind gravy<br>½ cup stir-fried cabbage<br>2 oz deep fried fish<br>½ cup sambar<br>1 small bowl rasam

DAY 2 (North Indian Menu)

	Conventional	Vegan	Ovo-vegetarian	Lacto-vegetarian	Lacto-ovo vegetarian	Plant-dominant
**Breakfast**	1 bowl vermicelli upma<br>2 tbsp peanuts<br>1 cup tea, 1 tbsp sugar	1 bowl vermicelli upma<br>2 tbsp peanuts	1 bowl vermicelli upma<br>2 tbsp peanuts	1 bowl vermicelli upma<br>2 tbsp peanuts	1 bowl vermicelli upma<br>2 tbsp peanuts	1 bowl vermicelli upma<br>2 tbsp peanuts
**Morning snack**	1 pc orange<br>1 cup tea, 1 tbsp sugar	1 pc orange	1 pc orange	1 pc orange	1 pc orange	1 pc orange
**Lunch**	1 medium bowl white rice<br>3 oz masala chicken<br>½ cup okra kadai sabji	1 medium bowl white rice<br>1½ cup masala chickpeas<br>½ cup okra kadai sabji	1 medium bowl white rice<br>2 pc masala egg<br>½ cup masala chickpeas<br>½ cup okra kadai sabji	1 medium bowl white rice<br>½ cup masala paneer<br>½ cup masala chickpeas<br>½ cup okra kadai sabji	1 medium bowl white rice<br>½ cup masala paneer<br>½ cup masala chickpeas<br>½ cup okra kadai sabji	1 medium bowl white rice<br>2 oz masala chicken<br>½ cup masala chickpeas<br>½ cup okra kadai sabji
**Evening snack**	1 slice papaya	1 slice papaya	1 slice papaya	1 slice papaya	1 slice papaya	1 slice papaya
**Dinner**	1 medium bowl white rice<br>3 oz manchurian/chili fish<br>½ cup curry eggplant<br>½ cup okra kadai sabji<br>1 cup tea, 1 tbsp sugar	1 medium bowl white rice<br>1½ cup chili lentils<br>½ cup curry eggplant<br>½ cup okra kadai sabji	1 medium bowl white rice<br>1½ cup chili lentils<br>½ cup curry eggplant<br>½ cup okra kadai sabji	1 medium bowl white rice<br>1½ cup chili lentils<br>½ cup curry eggplant<br>½ cup okra kadai sabji	1 medium bowl white rice<br>2 pc chili egg<br>½ cup chili lentils<br>½ cup curry eggplant<br>½ cup okra kadai sabji	1 medium bowl white rice<br>1 oz manchurian/chili fish<br>½ cup chili lentils<br>½ cup curry eggplant<br>½ cup okra kadai sabji

DAY 3 (South Chinese Menu)

Conventional	Vegan	Ovo-vegetarian	Lacto-vegetarian	Lacto-ovo vegetarian	Plant-dominant
**Breakfast**<br>Rice noodles with pork<br>1 medium bowl rice noodles<br>3 oz minced pork<br>¼ cup bok choy<br>1 cup tea, 1 tbsp sugar	**Breakfast**<br>Rice noodles with tofu<br>1 medium bowl rice noodles<br>2 pc tofu<br>¼ cup bok choy	**Breakfast**<br>Rice noodles with tofu<br>1 medium bowl rice noodles<br>1 pc tofu<br>¼ cup bok choy	**Breakfast**<br>Rice noodles with tofu<br>1 medium bowl rice noodles<br>1 pc tofu<br>¼ cup bok choy<br>1 glass low fat milk	**Breakfast**<br>Rice noodles with tofu<br>1 medium bowl rice noodles<br>1 pc tofu<br>¼ cup bok choy<br>1 glass low fat milk	**Breakfast**<br>Rice noodles with tofu<br>1 medium bowl rice noodles<br>1 pc tofu<br>¼ cup bok choy
**Morning snack**<br>1 pc pear<br>1 cup tea, 1 tbsp sugar	**Morning snack**<br>1 pc pear<br>1 glass soybean milk	**Morning snack**<br>1 pc pear<br>1 glass soybean milk	**Morning snack**<br>1 pc pear<br>½ glass low fat milk	**Morning snack**<br>1 pc pear<br>½ glass low fat milk	**Morning snack**<br>1 pc pear<br>1 glass soybean milk
**Lunch**<br>1 medium bowl white rice<br>2 oz steamed fish<br>½ cup cucumber, stir-fried<br>1 cup tea, 1 tbsp sugar	**Lunch**<br>1 medium bowl white rice<br>2 pc steamed tofu<br>½ cup cucumber, stir-fried	**Lunch**<br>1 medium bowl white rice<br>2 pc steamed tofu<br>½ cup cucumber, stir-fried	**Lunch**<br>1 medium bowl white rice<br>2 pc steamed tofu<br>½ cup cucumber, stir-fried	**Lunch**<br>1 medium bowl white rice<br>2 pc steamed tofu<br>½ cup cucumber, stir-fried	**Lunch**<br>1 medium bowl white rice<br>1 oz steamed fish<br>1 pc steamed tofu<br>½ cup cucumber, stir-fried
**Evening snack**<br>1 pc apple	**Evening snack**<br>1 pc apple	**Evening snack**<br>1 pc apple	**Evening snack**<br>1 pc apple<br>½ glass low fat milk	**Evening snack**<br>1 pc apple<br>½ glass low fat milk	**Evening snack**<br>1 pc apple
**Dinner**<br>1 medium bowl white rice<br>2 oz sesame oil chicken sauce<br>½ cup carrot, stir-fried<br>½ cup spinach, stir-fried<br>1 cup tea, 1 tbsp sugar	**Dinner**<br>1 medium bowl white rice<br>2 pc deep fried tofu<br>½ cup carrot, stir-fried<br>½ cup spinach, stir-fried	**Dinner**<br>1 medium bowl white rice<br>2 pc fried egg<br>1 pc fried tofu<br>½ cup carrot, stir-fried<br>½ cup spinach, stir-fried	**Dinner**<br>1 medium bowl white rice<br>2 pc steamed tofu<br>½ cup carrot, stir-fried<br>½ cup spinach, stir-fried	**Dinner**<br>1 medium bowl white rice<br>2 pc fried egg<br>1 pc steamed tofu<br>½ cup carrot, stir-fried<br>½ cup spinach, stir-fried	**Dinner**<br>1 medium bowl white rice<br>1 pc steamed tofu<br>1 oz steamed chicken<br>½ cup carrot, stir-fried<br>½ cup spinach, stir-fried

*Sugar may be replaced with honey, syrup, candies, or jam as other food choices.

Sample Menu for Moderate Protein Intake – 2100 kcal, 1.1 g/kg/d or 77 g/d protein
DAY 1 (South Indian Menu)

Conventional	Vegan	Ovo-vegetarian	Lacto-vegetarian	Lacto-ovo vegetarian	Plant-dominant
**Breakfast**<br>3 pc dosa<br>2 tbsp curry gravy<br>½ cup sambar	**Breakfast**<br>3 pc dosa<br>2 tbsp curry gravy<br>½ cup sambar	**Breakfast**<br>3 pc dosa<br>2 tbsp curry gravy<br>½ cup sambar	**Breakfast**<br>3 pc dosa<br>2 tbsp curry gravy<br>½ cup sambar	**Breakfast**<br>3 pc dosa<br>2 tbsp curry gravy<br>½ cup sambar	**Breakfast**<br>3 pc dosa<br>2 tbsp curry gravy<br>½ cup sambar
**Morning snack**<br>3 pc cream crackers	**Morning snack**<br>3 pc cream crackers	**Morning snack**<br>3 pc cream crackers	**Morning snack**<br>3 pc cream crackers<br>½ cup diluted buttermilk	**Morning snack**<br>3 pc cream crackers<br>½ cup diluted buttermilk	**Morning snack**<br>3 pc cream crackers
**Lunch**<br>1 medium bowl white rice<br>½ cup okra in tamarind gravy<br>½ cup french beans & carrot<br>Chicken curry:<br>2 oz chicken<br>2 tbsp curry gravy<br>1 small bowl rasam<br>½ pc mango	**Lunch**<br>1 medium bowl white rice<br>½ cup okra in tamarind gravy<br>½ cup french beans & carrot<br>Chickpeas curry:<br>1½ cup chickpea<br>2 tbsp curry gravy<br>1 small bowl rasam<br>½ pc mango	**Lunch**<br>1 medium bowl white rice<br>½ cup okra in tamarind gravy<br>½ cup french beans & carrot<br>Chickpeas curry:<br>1 cup chickpea<br>2 tbsp curry gravy<br>1 small bowl rasam<br>½ pc mango	**Lunch**<br>1 medium bowl white rice<br>½ cup okra in tamarind gravy<br>½ cup french beans & carrot<br>Chickpeas curry:<br>1 cup chickpea<br>2 tbsp curry gravy<br>½ cup curd<br>½ pc mango	**Lunch**<br>1 medium bowl white rice<br>½ cup okra in tamarind gravy<br>½ cup french beans & carrot<br>Chickpeas curry:<br>1 cup chickpea<br>2 tbsp curry gravy<br>½ cup curd<br>½ pc mango	**Lunch**<br>1 medium bowl white rice<br>½ cup okra in tamarind gravy<br>½ cup french beans & carrot<br>Chicken and chickpea curry:<br>1 oz chicken<br>½ cup chickpea<br>2 tbsp curry gravy<br>1 small bowl rasam<br>½ pc mango
**Evening snack**<br>1 pc apple	**Evening snack**<br>1 pc apple	**Evening snack**<br>1 pc apple	**Evening snack**<br>1 pc apple	**Evening snack**<br>1 pc apple	**Evening snack**<br>1 pc apple
**Dinner**<br>1 medium bowl white rice<br>¼ cup okra in tamarind gravy<br>½ cup stir-fried cabbage<br>3 oz pan fried fish<br>1 small bowl rasam	**Dinner**<br>1 medium bowl white rice<br>¼ cup okra in tamarind gravy<br>½ cup stir-fried cabbage<br>1½ cup sambar<br>1 small bowl rasam	**Dinner**<br>1 medium bowl white rice<br>¼ cup okra in tamarind gravy<br>½ cup stir-fried cabbage<br>2 pc fried egg<br>½ cup sambar<br>1 small bowl rasam	**Dinner**<br>1 medium bowl white rice<br>¼ cup okra in tamarind gravy<br>½ cup stir-fried cabbage<br>1 cup sambar<br>½ cup curd	**Dinner**<br>1 medium bowl white rice<br>¼ cup okra in tamarind gravy<br>½ cup stir-fried cabbage<br>2 pc fried egg<br>½ cup curd	**Dinner**<br>1 medium bowl white rice<br>¼ cup okra in tamarind gravy<br>½ cup stir-fried cabbage<br>2 oz pan-fried fish<br>½ cup sambar<br>1 small bowl rasam

DAY 2 (North Indian Menu)

Conventional	Vegan	Ovo-vegetarian	Lacto-vegetarian	Lacto-ovo vegetarian	Plant-dominant
**Breakfast**<br>1 bowl vermicelli upma<br>2 pc egg white<br>1 cup tea, 1 tbsp sugar	**Breakfast**<br>1 bowl vermicelli upma<br>2 tbsp peanuts	**Breakfast**<br>1 bowl vermicelli upma<br>2 tbsp peanuts<br>2 pc egg white	**Breakfast**<br>1 bowl vermicelli upma<br>2 tbsp peanuts	**Breakfast**<br>1 bowl vermicelli upma<br>2 tbsp peanuts<br>2 pc egg white	**Breakfast**<br>1 bowl vermicelli upma<br>2 tbsp peanuts
**Morning snack**<br>1 pc orange<br>1 cup tea, 1 tbsp sugar	**Morning snack**<br>1 pc orange	**Morning snack**<br>1 pc orange	**Morning snack**<br>1 pc orange	**Morning snack**<br>1 pc orange	**Morning snack**<br>1 pc orange
**Lunch**<br>1 medium bowl white rice<br>3 oz masala chicken<br>½ cup okra kadai sabji<br>1 cup tea, 1 tbsp sugar	**Lunch**<br>1 medium bowl white rice<br>2 cup masala chickpeas<br>½ cup okra kadai sabji	**Lunch**<br>1 medium bowl white rice<br>2 pc masala egg<br>½ cup masala chickpeas<br>½ cup okra kadai sabji	**Lunch**<br>1 medium bowl white rice<br>½ cup masala paneer<br>1 cup masala chickpeas<br>½ cup okra kadai sabji	**Lunch**<br>1 medium bowl white rice<br>½ cup masala paneer<br>½ cup masala chickpeas<br>½ cup okra kadai sabji	**Lunch**<br>1 medium bowl white rice<br>2 oz masala chicken<br>½ cup masala chickpeas<br>½ cup okra kadai sabji
**Evening snack**<br>1 slice papaya	**Evening snack**<br>1 slice papaya	**Evening snack**<br>1 slice papaya	**Evening snack**<br>1 slice papaya	**Evening snack**<br>1 slice papaya	**Evening snack**<br>1 slice papaya
**Dinner**<br>1 medium bowl white rice<br>3 oz manchurian/chili fish<br>½ cup curry eggplant<br>½ cup okra kadai sabji<br>1 cup tea, 1 tbsp sugar	**Dinner**<br>1 medium bowl white rice<br>1½ cup chili lentils<br>½ cup curry eggplant<br>½ cup okra kadai sabji	**Dinner**<br>1 medium bowl white rice<br>1 cup chili lentils<br>½ cup curry eggplant<br>½ cup okra kadai sabji	**Dinner**<br>1 medium bowl white rice<br>1½ cup chili lentils<br>½ cup curry eggplant<br>½ cup okra kadai sabji	**Dinner**<br>1 medium bowl white rice<br>2 pc chili egg<br>½ cup chili lentils<br>½ cup curry eggplant<br>½ cup okra kadai sabji	**Dinner**<br>1 medium bowl white rice<br>2 oz chili fish<br>1 cup chili lentils<br>½ cup curry eggplant<br>½ cup okra kadai sabji

DAY 3 (South Chinese Menu)

Conventional	Vegan	Ovo-vegetarian	Lacto-vegetarian	Lacto-ovo vegetarian	Plant-dominant
**Breakfast**<br>Rice noodles with pork<br>1 medium bowl rice noodles<br>3 oz minced pork<br>¼ cup bok choy<br>1 cup tea, 1 tbsp sugar	**Breakfast**<br>Rice noodles with tofu<br>1 medium bowl rice noodles<br>2 pc tofu<br>¼ cup bok choy	**Breakfast**<br>Rice noodles with tofu<br>1 medium bowl rice noodles<br>2 pc tofu<br>¼ cup bok choy	**Breakfast**<br>Rice noodles with tofu<br>1 medium bowl rice noodles<br>2 pc tofu<br>¼ cup bok choy<br>1 glass low fat milk	**Breakfast**<br>Rice noodles with tofu<br>1 medium bowl rice noodles<br>2 pc tofu<br>¼ cup bok choy<br>1 glass low fat milk	**Breakfast**<br>Rice noodles with tofu<br>1 medium bowl rice noodles<br>1 pc tofu<br>¼ cup bok choy
**Morning snack**<br>1 pc pear<br>1 cup tea, 1 tbsp sugar	**Morning snack**<br>1 pc pear<br>1 glass soybean milk	**Morning snack**<br>1 pc pear<br>1 glass soybean milk	**Morning snack**<br>1 pc pear<br>½ glass low fat milk	**Morning snack**<br>1 pc pear<br>½ glass low fat milk	**Morning snack**<br>1 pc pear<br>1 glass soybean milk
**Lunch**<br>1 medium bowl white rice<br>2 oz steamed fish<br>½ cup cucumber, stir-fried<br>1 cup tea, 1 tbsp sugar	**Lunch**<br>1 medium bowl white rice<br>2 pc steamed tofu<br>½ cup cucumber, stir-fried	**Lunch**<br>1 medium bowl white rice<br>2 pc steamed tofu<br>½ cup cucumber, stir-fried	**Lunch**<br>1 medium bowl white rice<br>2 pc steamed tofu<br>½ cup cucumber, stir-fried	**Lunch**<br>1 medium bowl white rice<br>2 pc steamed tofu<br>½ cup cucumber, stir-fried	**Lunch**<br>1 medium bowl white rice<br>1 oz steamed fish<br>2 pc steamed tofu<br>½ cup cucumber, stir-fried
**Evening snack**<br>1 pc apple	**Evening snack**<br>1 pc apple	**Evening snack**<br>1 pc apple	**Evening snack**<br>1 pc apple<br>½ glass low fat milk	**Evening snack**<br>1 pc apple<br>½ glass low fat milk	**Evening snack**<br>1 pc apple
**Dinner**<br>1 medium bowl white rice<br>3 oz steamed chicken<br>½ cup carrot, stir-fried<br>½ cup spinach, stir-fried<br>1 cup tea, 1 tbsp sugar	**Dinner**<br>1 medium bowl white rice<br>2⅔ pc steamed tofu<br>½ cup carrot, stir-fried<br>½ cup spinach, stir-fried	**Dinner**<br>1 medium bowl white rice<br>2 pc fried egg<br>1 1/3 pc fried tofu<br>½ cup carrot, stir-fried<br>½ cup spinach, stir-fried	**Dinner**<br>1 medium bowl white rice<br>2 pc steamed tofu<br>½ cup carrot, stir-fried<br>½ cup spinach, stir-fried	**Dinner**<br>1 medium bowl white rice<br>2 pc fried egg<br>1 pc steamed tofu<br>½ cup carrot, stir-fried<br>½ cup spinach, stir-fried	**Dinner**<br>1 medium bowl white rice<br>1 pc steamed tofu<br>1 oz steamed chicken<br>½ cup carrot, stir-fried<br>½ cup spinach, stir-fried

*Sugar may be replaced with honey, syrup, candies, or jam as other food choices.

Sample Menu for Moderate Protein Intake – 2100 kcal, 1.2 g/kg/d or 84 g/d protein

DAY 1 (South Indian Menu)

Conventional	Vegan	Ovo-vegetarian	Lacto-vegetarian	Lacto-ovo vegetarian	Plant-dominant
**Breakfast**<br>3 pc dosa<br>2 tbsp curry gravy<br>½ cup sambar	**Breakfast**<br>3 pc dosa<br>2 tbsp curry gravy<br>1 cup sambar	**Breakfast**<br>3 pc dosa<br>2 tbsp curry gravy<br>½ cup sambar	**Breakfast**<br>3 pc dosa<br>2 tbsp curry gravy<br>½ cup sambar	**Breakfast**<br>3 pc dosa<br>2 tbsp curry gravy<br>½ cup sambar	**Breakfast**<br>3 pc dosa<br>2 tbsp curry gravy<br>½ cup sambar
**Morning snack**<br>3 pc cream crackers	**Morning snack**<br>3 pc cream crackers	**Morning snack**<br>3 pc cream crackers	**Morning snack**<br>3 pc cream crackers<br>½ cup diluted buttermilk	**Morning snack**<br>3 pc cream crackers<br>½ cup diluted buttermilk	**Morning snack**<br>3 pc cream crackers
**Lunch**<br>1 medium bowl white rice<br>½ cup okra in tamarind gravy<br>½ cup french beans & carrot<br>Chicken curry:<br>3 oz chicken<br>2 tbsp curry gravy<br>1 small bowl rasam<br>½ pc mango	**Lunch**<br>1 medium bowl white rice<br>½ cup okra in tamarind gravy<br>½ cup french beans & carrot<br>Chickpeas curry:<br>1½ cup chickpea<br>2 tbsp curry gravy<br>1 small bowl rasam<br>½ pc mango	**Lunch**<br>1 medium bowl white rice<br>½ cup okra in tamarind gravy<br>½ cup french beans & carrot<br>Chickpeas curry:<br>1 ½ cup chickpea<br>2 tbsp curry gravy<br>1 small bowl rasam<br>½ pc mango	**Lunch**<br>1 medium bowl white rice<br>½ cup okra in tamarind gravy<br>½ cup french beans & carrot<br>Chickpeas curry:<br>1 cup chickpea<br>2 tbsp curry gravy<br>½ cup curd<br>½ pc mango	**Lunch**<br>1 medium bowl white rice<br>½ cup okra in tamarind gravy<br>½ cup french beans & carrot<br>Chickpeas curry:<br>1 cup chickpea<br>2 tbsp curry gravy<br>½ cup curd<br>½ pc mango	**Lunch**<br>1 medium bowl white rice<br>½ cup okra in tamarind gravy<br>½ cup french beans & carrot<br>Chicken and chickpea curry:<br>1 oz chicken<br>½ cup chickpea<br>2 tbsp curry gravy<br>1 small bowl rasam<br>½ pc mango
**Evening snack**<br>1 pc apple	**Evening snack**<br>1 pc apple	**Evening snack**<br>1 pc apple	**Evening snack**<br>1 pc apple	**Evening snack**<br>1 pc apple	**Evening snack**<br>1 pc apple
**Dinner**<br>1 medium bowl white rice<br>¼ cup okra in tamarind gravy<br>½ cup stir-fried cabbage<br>3 oz pan fried fish<br>1 small bowl rasam<br>1 cup tea, 1 tbsp sugar	**Dinner**<br>1 medium bowl white rice<br>¼ cup okra in tamarind gravy<br>½ cup stir-fried cabbage<br>1½ cup sambar<br>1 small bowl rasam	**Dinner**<br>1 medium bowl white rice<br>¼ cup okra in tamarind gravy<br>½ cup stir-fried cabbage<br>2 pc fried egg<br>½ cup sambar<br>1 small bowl rasam	**Dinner**<br>1 medium bowl white rice<br>¼ cup okra in tamarind gravy<br>½ cup stir-fried cabbage<br>1½ cup sambar<br>½ cup curd	**Dinner**<br>1 medium bowl white rice<br>¼ cup okra in tamarind gravy<br>½ cup stir-fried cabbage<br>2 pc fried egg<br>½ cup sambar<br>½ cup curd	**Dinner**<br>1 medium bowl white rice<br>¼ cup okra in tamarind gravy<br>½ cup stir-fried cabbage<br>2 oz pan-fried fish<br>1 cup sambar<br>1 small bowl rasam

DAY 2 (North Indian Menu)

	Conventional	Vegan	Ovo-vegetarian	Lacto-vegetarian	Lacto-ovo vegetarian	Plant-dominant
**Breakfast**	1 bowl vermicelli upma<br>2 pc egg white<br>1 cup tea, 1 tbsp sugar	1 bowl vermicelli upma<br>2 tbsp peanuts	1 bowl vermicelli upma<br>2 tbsp peanuts<br>2 pc egg white	1 bowl vermicelli upma<br>2 tbsp peanuts	1 bowl vermicelli upma<br>2 tbsp peanuts<br>2 pc egg white	1 bowl vermicelli upma<br>2 tbsp peanuts
**Morning snack**	1 pc orange<br>1 cup tea, 1 tbsp sugar	1 pc orange	1 pc orange	1 pc orange	1 pc orange	1 pc orange
**Lunch**	1 medium bowl white rice<br>3½ oz masala chicken<br>½ cup okra kadai sabji<br>1 cup tea, 1 tbsp sugar	1 medium bowl white rice<br>2 cup masala chickpeas<br>½ cup okra kadai sabji	1 medium bowl white rice<br>2 pc masala egg<br>1 cup masala chickpeas<br>½ cup okra kadai sabji	1 medium bowl white rice<br>½ cup masala paneer<br>1½ cup masala chickpeas<br>½ cup okra kadai sabji	1 medium bowl white rice<br>½ cup masala paneer<br>1 cup masala chickpeas<br>½ cup okra kadai sabji	1 medium bowl white rice<br>2 oz masala chicken<br>1 cup masala chickpeas<br>½ cup okra kadai sabji
**Evening snack**	1 slice papaya	1 slice papaya	1 slice papaya	1 slice papaya	1 slice papaya	1 slice papaya<br>2 tbsp peanuts
**Dinner**	1 medium bowl white rice<br>3½ oz manchurian/chili fish<br>½ cup curry eggplant<br>½ cup okra kadai sabji<br>1 cup tea, 1 tbsp sugar	1 medium bowl white rice<br>2 cup chili lentils<br>½ cup curry eggplant<br>½ cup okra kadai sabji	1 medium bowl white rice<br>1½ cup chili lentils<br>½ cup curry eggplant<br>½ cup okra kadai sabji	1 medium bowl white rice<br>1½ cup chili lentils<br>½ cup curry eggplant<br>½ cup okra kadai sabji	1 medium bowl white rice<br>2 pc chili egg<br>½ cup chili lentils<br>½ cup curry eggplant<br>½ cup okra kadai sabji	1 medium bowl white rice<br>2 oz chili fish<br>1 cup chili lentils<br>½ cup curry eggplant<br>½ cup okra kadai sabji

DAY 3 (South Chinese Menu)

	Conventional	Vegan	Ovo-vegetarian	Lacto-vegetarian	Lacto-ovo vegetarian	Plant-dominant
**Breakfast**	Rice noodles with pork<br>1 medium bowl rice noodles<br>3 oz minced pork<br>¼ cup bok choy<br>1 cup tea, 1 tbsp sugar	Rice noodles with tofu<br>1 medium bowl rice noodles<br>2 pc tofu<br>¼ cup bok choy	Rice noodles with tofu<br>1 medium bowl rice noodles<br>2 pc tofu<br>¼ cup bok choy	Rice noodles with tofu<br>1 medium bowl rice noodles<br>2 pc tofu<br>¼ cup bok choy<br>1 glass low fat milk	Rice noodles with tofu<br>1 medium bowl rice noodles<br>2 pc tofu<br>¼ cup bok choy<br>1 glass low fat milk	Rice noodles with tofu<br>1 medium bowl rice noodles<br>1 pc tofu<br>¼ cup bok choy
**Morning snack**	1 pc pear<br>1 cup tea, 1 tbsp sugar	1 pc pear<br>1 glass soybean milk	1 pc pear<br>1 glass soybean milk	1 pc pear<br>½ glass low fat milk	1 pc pear<br>½ glass low fat milk	1 pc pear<br>1 glass soybean milk
**Lunch**	1 medium bowl white rice<br>3 oz steamed fish<br>½ cup cucumber, stir-fried<br>1 cup tea, 1 tbsp sugar	1 medium bowl white rice<br>2⅔ pc steamed tofu<br>½ cup cucumber, stir-fried	1 medium bowl white rice<br>2⅔ pc steamed tofu<br>½ cup cucumber, stir-fried	1 medium bowl white rice<br>2⅔ pc steamed tofu<br>½ cup cucumber, stir-fried	1 medium bowl white rice<br>2 pc steamed tofu<br>½ cup cucumber, stir-fried	1 medium bowl white rice<br>2 oz steamed fish<br>2 pc steamed tofu<br>½ cup cucumber, stir-fried
**Evening snack**	1 pc apple	1 pc apple	1 pc apple	1 pc apple<br>½ glass low fat milk	1 pc apple<br>½ glass low fat milk	1 pc apple
**Dinner**	1 medium bowl white rice<br>3 oz steamed chicken<br>½ cup carrot, stir-fried<br>½ cup spinach, stir-fried<br>1 cup tea, 1 tbsp sugar	1 medium bowl white rice<br>2⅔ pc steamed tofu<br>½ cup carrot, stir-fried<br>½ cup spinach, stir-fried	1 medium bowl white rice<br>2 pc fried egg<br>1 pc fried tofu<br>½ cup carrot, stir-fried<br>½ cup spinach, stir-fried	1 medium bowl white rice<br>2⅔ pc steamed tofu<br>½ cup carrot, stir-fried<br>½ cup spinach, stir-fried	1 medium bowl white rice<br>2 pc fried egg<br>2 pc steamed tofu<br>½ cup carrot, stir-fried<br>½ cup spinach, stir-fried<br>1 cup tea, 1 tbsp sugar	1 medium bowl white rice<br>1 pc steamed tofu<br>1 oz steamed chicken<br>½ cup carrot, stir-fried<br>½ cup spinach, stir-fried

*Sugar may be replaced with honey, syrup, candies, or jam as other food choices.
